# The $N$-Link Swimmer in Three Dimensions: Controllability and Optimality Results

**DOI:** 10.1007/s10440-022-00480-3

**Published:** 2022-03-08

**Authors:** Roberto Marchello, Marco Morandotti, Henry Shum, Marta Zoppello

**Affiliations:** 1 Dipartimento di Scienze Matematiche “G. L. Lagrange”, Politecnico di Torino, Corso Duca degli Abruzzi, 24, 10129 Torino, Italy; 2 Department of Applied Mathematics, University of Waterloo, 200 University Avenue West, Waterloo, ON Canada N2L 3G1

**Keywords:** Motion in viscous fluids, Micro-swimmers, Resistive force theory, Controllability, Optimal control problems, 93B05, 76Z10, 70Q05, 93C10, 49J15

## Abstract

The controllability of a fully three-dimensional $N$-link swimmer is studied. After deriving the equations of motion in a low Reynolds number fluid by means of Resistive Force Theory, the controllability of the minimal 2-link swimmer is tackled using techniques from Geometric Control Theory. The shape of the 2-link swimmer is described by two angle parameters. It is shown that the associated vector fields that govern the dynamics generate, via taking their Lie brackets, all eight linearly independent directions in the combined configuration and shape space, leading to controllability; the swimmer can move from any starting configuration and shape to any target configuration and shape by operating on the two shape variables. The result is subsequently extended to the $N$-link swimmer. Finally, the minimal time optimal control problem and the minimization of the power expended are addressed and a qualitative description of the optimal strategies is provided.

## Introduction

The swimming motion of microorganisms in viscous fluids at low Reynolds number has been studied mathematically since the 1950s [22, 39]. There has recently been growing interest in understanding the behavior of simple model swimmers due to the potential to manufacture such microrobots and use them for biomedical applications [29, 38]. For practical reasons, it can be beneficial for a proposed robotic swimmer to be as simple as possible while achieving full controllability. Here, we define swimming to be the translational and rotational motion of the swimmer in quiescent fluid due to changes in shape of the swimmer’s body; by controllability we mean the ability of prescribing the shape changes in order to steer the swimmer from a given initial configuration (*i.e.*, position and orientation) to a given final one. We neglect gravity, assuming that the swimmer is neutrally buoyant, and in view of proposing a model for a minimal swimmer, other net forces and torques acting on the body are not considered.

It is well known for swimmers in Stokes flow that if the body undergoes a shape change that is subsequently reversed, then the swimmer would return to its original position and orientation. This result, stated by Purcell [35], is known as the Scallop Theorem. In particular, a “scallop” consisting of two rigid links joined by a hinge that can open and close will not achieve any net displacement by repeatedly opening and closing its hinge. Purcell proposed that at least three links, connected by two hinges, are necessary to achieve a net displacement with periodic shape changes. This model is commonly referred to as Purcell’s (planar) 3-link swimmer, and has been shown to be controllable in two-dimensional space [17, 27].

If the 3-link swimmer is twisted so that the axes of rotation for the two hinges are perpendicular to one another, then the swimmer is no longer planar in configuration. While this variant still has only two hinges, and therefore two degrees of freedom for the shape, it was shown that this swimmer is controllable in three-dimensional space [20].

In the present work, we consider a 2-link swimmer that has a joint with two angular degrees of freedom. This joint can be thought of as a hinge whose axis can rotate about the axis of the first link. Alternatively, this corresponds to the non-planar 3-link swimmer in the limit that the length of the central link vanishes so that the two perpendicular hinges are next to each other.

Note that there is a fundamental difference between the 2-link swimmer with two degrees of motion and the non-planar 3-link swimmer. It is clear that opening or closing either hinge changes the shape of the 3-link swimmer. Without the central link, however, one of the hinges simply rotates a link about its axis. To an outside observer, the shape appears indistinguishable since each link is assumed to be a cylinder with rotational symmetry. Nevertheless, we show that the 2-link swimmer can achieve arbitrary displacements and rotations in three-dimensional space provided that the model incorporates a viscous torque due to rotation of one of the links about its axis. This torque enables the swimmer to rotate despite its shape appearing stationary due to the symmetry of the cylindrical link.

**Fig. 1:**
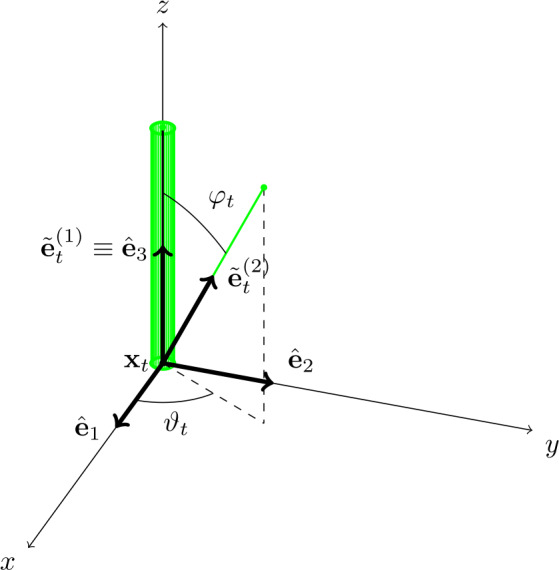
In black, the co-moving frame of the 2-link swimmer; in green, the swimmer itself, with the thicker link 1 aligned with the $z$ axis. (Color figure online)

Our 2-link swimmer consists of two thin, cylindrical rods (the links) connected end-to-end by a joint that allows arbitrary rotation of the rods relative to one another (see Fig. 1). We associate the co-moving frame of the swimmer with the first link such that link 1 is oriented in the positive $z$ direction of this reference frame and the joint is at the origin. The orientation of the second link in this reference frame is described by two angles $\vartheta $ and $\varphi $, which parametrize a point in $S^{2}$. The angles $\vartheta $ and $\varphi $ are the shape parameters of the swimmer. The configuration parameters are the translation $x\in R^{3}$ and rotation $R\in SO(3)$ of the change of coordinates of the co-moving frame with respect to the laboratory reference frame.

By the considerations above, the only forces acting on the swimmer are the hydrodynamic ones which, due to the slenderness of the swimmer, can be accurately approximated by Resistive Force Theory [19]. Under this approximation, we consider a one-dimensional distribution of viscous force density acting along the centerline of each link. The magnitudes of the force per unit length in the parallel and perpendicular directions are proportional to the parallel and perpendicular components of the local velocity, respectively. Integrating the moment of the viscous force yields a viscous torque due to rotations of the link about an axis perpendicular to the link.

No torque arises from viscous forces when a link rotates about its axis, however, because the centerline has zero linear velocity. To ensure that the rotational velocity of the co-moving frame is uniquely defined, we include a torque on the first link that is proportional to the rotational velocity of the link about its axis. Without this torque, an arbitrary rotation rate about the axis of link 1 could be added without affecting the dynamics.

Note that we do not need to include a similar torque on the second link because we consider only the direction of the second link, described by two angles, as the shape parameters. We do not have a third shape parameter to track rotations of the second link about its own axis.

Once the total viscous force and torque are computed, setting them equal to zero allows us to obtain the equations of motion for the swimmer. These are conveniently written in the form of a (nonlinear) control system, so that tools from Geometric Control Theory can be applied. In this framework, the time changes of the shape parameters $\vartheta $ and $\varphi $ are considered as the controls $u_{1}$, $u_{2}$ of the system, since $\vartheta $ and $\varphi $ are the parameters that can be actuated by the swimmer to modify its shape. Standard results and methods from Geometric Control Theory are used to prove Theorem 3.6 ensuring controllability of the 2-link swimmer: any given final configuration can be reached starting from any assigned initial configuration by acting on the controls $u_{1}$, $u_{2}$. Technically, this is obtained by computing the Lie brackets of the vector fields $V_{1}$ and $V_{2}$ activated by $u_{1}$ and $u_{2}$ and showing that they generate all the possible directions of motion, thus proving that two linearly independent vectors, the $V_{i}$’s, generate the eight-dimensional space of translations, rotations, and shapes $(\mathbf{x},R,(\varphi ,\vartheta ))$. This strategy was introduced in [5, 17] and then generalized in [9, 10, 31].

Controllability for the 2-link both ensures that the equations of motion have a unique solution (Theorem 2.2) and can be easily extended to the $N$-link swimmer, providing the main result of the paper.

Controllability of the system paves the way to the study of optimal swimming strategies. Our second result establishes the existence of an optimal solution and the qualitative characterization of the optimal control that generates it, for two specific optimal control problems which are relevant for the applications, especially in view of possible robotic implementations. The minimal time optimal control problem seeks the optimal solution to move from a given configuration to another given one in the shortest possible time, whereas optimization of the power expended deals with minimizing the power expended to achieve the motion (this is useful in consideration of a limited amount of resources). Similar optimal control problems have been tackled in [9, 13, 17, 30, 32] for the power expended of a filament moving on a plane, for the minimal time of a planar Purcell swimmer, and for the minimal time ad quadratic cost for a scallop subject to a switching dynamics, respectively.

The paper is organized as follows: in Sect. 2 we describe the setting for the dynamics of the 2-link swimmer, and we deduce the equations of motion. In Sect. 3, we prove Theorem 3.6 which states that the 2-link swimmer is controllable. We further describe how to generalize both the problem setting and the results obtained to the $N$-link swimmer in Sect. 4 and, in Sect. 5, we discuss some optimal control problems which are relevant in this context, namely the minimal time optimal control problem and the minimization of the power expended. Finally, Sect. 6 collects an overview of the results obtained and discusses some potential perspectives.

## Dynamics of the 2-Link Swimmer

The formulation and analysis of the swimmer model follow those in previous studies of planar $N$-link swimmers [2, 27], but the extension to three-dimensional motion introduces additional complexity to the equations and notation.

Let $x_{t}\in R^{3}$ denote the position at time $t$ of the origin of the reference frame that moves with the swimmer with respect to the stationary laboratory frame and let $R_{t}\in SO(3)$ be the rotation matrix whose columns are the orthonormal basis vectors, $\hat{\mathbf {e}}_{i}$, $i\in \{1,2,3\}$, of the co-moving frame. Below, we use tildes to indicate vectors expressed in the co-moving frame whereas vectors without tildes are in the laboratory frame. A generic direction vector $\mathbf {v}_{t}$ (unaffected by translations of the origin) transforms according to

2.1 $$ \mathbf {v}_{t} = R_{t}\tilde{\mathbf {v}}_{t}. $$

We place the joint of the 2-link swimmer at the origin of the co-moving frame and set the direction $\mathbf {e}_{t}^{(1)}$ of the first link to be the positive $\hat{\mathbf {e}}_{3}$ direction, i.e., $\tilde{\mathbf {e}}_{t}^{(1)} = (0,0,1)$ for all $t$. The unit direction vector of the second link is denoted by $\tilde{\mathbf {e}}_{t}^{(2)}$ in the moving frame and by $\mathbf {e}_{t}^{(2)} = R_{t} \tilde{\mathbf {e}}_{t}^{(2)}$ in the stationary frame. We use the spherical polar coordinate system in the co-moving frame to define

2.2 $$ \tilde{\mathbf {e}}^{(2)}_{t}:=(\sin \varphi _{t}\cos \vartheta _{t},\sin \varphi _{t}\sin \vartheta _{t},\cos \varphi _{t}), $$

where $(\varphi _{t},\vartheta _{t})\in (0,\pi )\times (-\pi ,\pi )$ are the *shape parameters* of the 2-link swimmer.

Finally, let $\ell _{i}$ be the length of the $i$-th link, so that a generic point on the $i$-th link, at a distance $s\in [0,\ell _{i}]$ from the joint is given by $\tilde{\mathbf {x}}_{t}^{(i)}(s)=s\tilde{\mathbf {e}}^{(i)}_{t}$ in the co-moving frame, and by

2.3 $$ \mathbf {x}^{(i)}_{t}(s)=\mathbf {x}_{t}+s\mathbf {e}^{(i)}_{t}=\mathbf {x}_{t}+sR_{t}\tilde{\mathbf {e}}^{(i)}_{t} $$

in the laboratory frame.

The densities of viscous force $\mathbf{f}^{(i)}_{t}(s)$ and torque $\boldsymbol{\tau }^{(i)}_{t}(s)$ are computed using *Resistive Force Theory* by

2.4a $$\begin{aligned} \mathbf{f}^{(i)}_{t}(s)= &\, [(C_{\parallel }-C_{\perp })\mathbf{e}^{(i)}_{t} \otimes \mathbf{e}^{(i)}_{t}+C_{\perp }I]\, \dot{\mathbf{x}}^{(i)}_{t}(s), \end{aligned}$$

2.4b $$\begin{aligned} \boldsymbol{\tau }^{(1)}_{t}(s)= &\, s\mathbf{e}^{(1)}_{t} \times \mathbf{f}^{(1)}_{t}(s)+C_{\tau }( \mathbf{e}^{(1)}_{t}\otimes \mathbf{e}^{(1)}_{t}) \boldsymbol{\omega }_{t}, \end{aligned}$$

2.4c $$\begin{aligned} \boldsymbol{\tau }^{(2)}_{t}(s)= &\, s\mathbf{e}^{(2)}_{t} \times \mathbf{f}^{(2)}_{t}(s), \end{aligned}$$

where $C_{\parallel }$ and $C_{\perp }$ are the parallel and perpendicular drag coefficients to each link and $C_{\tau }$ is the torsional drag coefficient which takes into account the fact that the first link is a cylinder, the symbol ⊗ denotes the dyadic product of vectors ($(\mathbf{a} \otimes \mathbf{b})_{ij}:=a_{i}b_{j}$), the symbol × denotes the vector product in $R^{3}$, and a superimposed dot denotes derivation with respect to time.

In order to compute the expressions in (2.4a)–(2.4c), we need to take the time derivative $\dot{\mathbf{x}}^{(i)}_{t}(s)$, which, by (2.1) and (2.3), reads

2.5 $$ \begin{aligned} \dot{\mathbf{x}}^{(i)}_{t}(s)=\dot{\mathbf{x}}_{t}+s \dot{\mathbf{e}}^{(i)}_{t}= & \,\dot{\mathbf{x}}_{t}+s\dot{R}_{t} \tilde{\mathbf{e}}^{(i)}_{t}+sR_{t}\dot{\tilde{\mathbf{e}}}^{(i)}_{t} \\ =&\, \dot{\mathbf{x}}_{t}+s\dot{R}_{t} R^{-1}_{t} R_{t} \tilde{\mathbf{e}}^{(i)}_{t}+sR_{t}\dot{\tilde{\mathbf{e}}}^{(i)}_{t} \\ =&\, \dot{\mathbf{x}}_{t}+s\Omega _{t} \mathbf{e}^{(i)}_{t}+sR_{t} \dot{\tilde{\mathbf{e}}}^{(i)}_{t} = \dot{\mathbf{x}}_{t}+s \boldsymbol{\omega }_{t} \times \mathbf{e}^{(i)}_{t}+sR_{t} \dot{\tilde{\mathbf{e}}}^{(i)}_{t} \,, \end{aligned} $$

where $\Omega _{t}$ and $\boldsymbol{\omega }_{t}$ are the angular matrix and the angular velocity, respectively, associated with the rotation matrix $R_{t}$.

Taking some elementary vector identities^1^ into account, we obtain

2.6 $$ \begin{aligned} \mathbf{f}^{(1)}_{t}(s)= &\, [(C_{\parallel }-C_{\perp }) \mathbf{e}^{(1)}_{t}\otimes \mathbf{e}^{(1)}_{t}+C_{\perp }I] \dot{\mathbf{x}}_{t}+sC_{\perp }\boldsymbol{\omega }_{t}\times \mathbf{e}^{(1)}_{t}\,, \\ \mathbf{f}^{(2)}_{t}(s)= &\, [(C_{\parallel }-C_{\perp })\mathbf{e}^{(2)}_{t} \otimes \mathbf{e}^{(2)}_{t}+C_{\perp }I]\dot{\mathbf{x}}_{t}+sC_{\perp }\boldsymbol{\omega }_{t}\times \mathbf{e}^{(2)}_{t}+sC_{\perp }R_{t} \dot{\tilde{\mathbf{e}}}^{(2)}_{t}\,, \end{aligned} $$

where we also used that $\lvert \tilde{\mathbf{e}}^{(i)}_{t}\rvert \equiv 1$ implies that $\tilde{\mathbf{e}}^{(i)}_{t}\cdot \dot{\tilde{\mathbf{e}}}^{(i)}_{t}=0$. Moreover,

$$ \begin{aligned} \boldsymbol{\tau }^{(1)}_{t}(s)= &\, sC_{\perp }\mathbf{e}^{(1)}_{t} \times \dot{\mathbf{x}}_{t}+s^{2}C_{\perp }[I-\mathbf{e}^{(1)}_{t} \otimes \mathbf{e}^{(1)}_{t}]\boldsymbol{\omega }_{t}+C_{\tau }( \mathbf{e}^{(1)}_{t}\otimes \mathbf{e}^{(1)}_{t}) \boldsymbol{\omega }_{t}\,, \\ \boldsymbol{\tau }^{(2)}_{t}(s)= &\, sC_{\perp }\mathbf{e}^{(2)}_{t} \times \dot{\mathbf{x}}_{t}+s^{2}C_{\perp }[I-\mathbf{e}^{(2)}_{t} \otimes \mathbf{e}^{(2)}_{t}]\boldsymbol{\omega }_{t}+s^{2}C_{\perp }\mathbf{e}^{(2)}_{t}\times R_{t}\dot{\tilde{\mathbf{e}}}^{(2)}_{t}\,. \end{aligned} $$

Integrating from 0 to $\ell _{i}$ with respect to $s$, we obtain

$$\begin{aligned} \mathbf{F}^{(1)}_{t} =&\, [(C_{\parallel }-C_{\perp }) \mathbf{e}^{(1)}_{t}\otimes \mathbf{e}^{(1)}_{t}+C_{\perp }I]\ell _{1} \dot{\mathbf{x}}_{t}+\frac{\ell _{1}^{2}}{2}C_{\perp }\boldsymbol{\omega }_{t}\times \mathbf{e}^{(1)}_{t} \\ =&\, \ell _{1} R_{t} [(C_{\parallel }-C_{\perp })\tilde{\mathbf{e}}^{(1)}_{t} \otimes \tilde{\mathbf{e}}^{(1)}_{t}+C_{\perp }I] R_{t}^{-1} \dot{\mathbf{x}}_{t} -\frac{\ell _{1}^{2}}{2}C_{\perp }R_{t} [ \tilde{\mathbf{e}}^{(1)}_{t}\times (R_{t}^{-1}\boldsymbol{\omega }_{t})], \\ \mathbf{F}^{(2)}_{t}= &\, [(C_{\parallel }-C_{\perp })\mathbf{e}^{(2)}_{t} \otimes \mathbf{e}^{(2)}_{t}+C_{\perp }I]\ell _{2}\dot{\mathbf{x}}_{t}+ \frac{\ell _{2}^{2}}{2}C_{\perp }\boldsymbol{\omega }_{t}\times \mathbf{e}^{(2)}_{t}+\frac{\ell _{2}^{2}}{2}C_{\perp }R_{t} \dot{\tilde{\mathbf{e}}}^{(2)}_{t} \\ =&\, \ell _{2} R_{t} [(C_{\parallel }-C_{\perp })\tilde{\mathbf{e}}^{(2)}_{t} \otimes \tilde{\mathbf{e}}^{(2)}_{t}+C_{\perp }I] R_{t}^{-1} \dot{\mathbf{x}}_{t}\\ & -\frac{\ell _{2}^{2}}{2}C_{\perp }R_{t} [ \tilde{\mathbf{e}}^{(2)}_{t}\times (R_{t}^{-1}\boldsymbol{\omega }_{t})] +\frac{\ell _{2}^{2}}{2}C_{\perp }R_{t}\dot{\tilde{\mathbf{e}}}^{(2)}_{t}, \\ \mathbf{T}^{(1)}_{t}= &\, \frac{\ell _{1}^{2}}{2}C_{\perp }\mathbf{e}^{(1)}_{t} \times \dot{\mathbf{x}}_{t}+\frac{\ell _{1}^{3}}{3}C_{\perp }[I- \mathbf{e}^{(1)}_{t}\otimes \mathbf{e}^{(1)}_{t}]\boldsymbol{\omega }_{t}+ \ell _{1}C_{\tau }( \mathbf{e}^{(1)}_{t}\otimes \mathbf{e}^{(1)}_{t}) \boldsymbol{\omega }_{t} \\ =&\, \frac{\ell _{1}^{2}}{2}C_{\perp }R_{t} [ \tilde{\mathbf{e}}^{(1)}_{t} \times (R_{t}^{-1} \dot{\mathbf{x}}_{t})]+\frac{\ell _{1}^{3}}{3}C_{\perp }R_{t}[I-\tilde{\mathbf{e}}^{(1)}_{t}\otimes \tilde{\mathbf{e}}^{(1)}_{t}] R_{t}^{-1}\boldsymbol{\omega }_{t} \\ & +\ell _{1}C_{\tau }R_{t}(\tilde{ \mathbf{e}}^{(1)}_{t}\otimes \tilde{\mathbf{e}}^{(1)}_{t}) R_{t}^{-1}\boldsymbol{\omega }_{t}, \\ \mathbf{T}^{(2)}_{t}= &\, \frac{\ell _{2}^{2}}{2}C_{\perp }\mathbf{e}^{(2)}_{t} \times \dot{\mathbf{x}}_{t}+\frac{\ell _{2}^{3}}{3}C_{\perp }[I- \mathbf{e}^{(2)}_{t}\otimes \mathbf{e}^{(2)}_{t}]\boldsymbol{\omega }_{t}+ \frac{\ell _{2}^{3}}{3}C_{\perp }\mathbf{e}^{(2)}_{t}\times R_{t} \dot{\tilde{\mathbf{e}}}^{(2)}_{t} \\ =&\, \frac{\ell _{2}^{2}}{2}C_{\perp }R_{t} [ \tilde{\mathbf{e}}^{(2)}_{t} \times (R_{t}^{-1} \dot{\mathbf{x}}_{t})]+\frac{\ell _{2}^{3}}{3}C_{\perp }R_{t}[I-\tilde{\mathbf{e}}^{(2)}_{t}\otimes \tilde{\mathbf{e}}^{(2)}_{t}] R_{t}^{-1}\boldsymbol{\omega }_{t}\\ & + \frac{\ell _{2}^{3}}{3}C_{\perp }R_{t} (\tilde{\mathbf{e}}^{(2)}_{t}\times \dot{\tilde{\mathbf{e}}}^{(2)}_{t}). \end{aligned}$$

The total viscous force is then given by

2.7 $$ \mathbf{F}_{t}=\mathbf{F}^{(1)}_{t}+\mathbf{F}^{(2)}_{t}= R_{t} \tilde{K}_{t}R_{t}^{-1}\dot{\mathbf{x}}_{t} + R_{t} \tilde{C}_{t}^{\top }R_{t}^{-1} \boldsymbol{\omega }_{t}+R_{t} \tilde{\mathbf{F}}^{\mathrm{sh}}_{t} $$

and the total viscous torque by

2.8 $$ \mathbf{T}_{t}=\mathbf{T}^{(1)}_{t}+\mathbf{T}^{(2)}_{t}= R_{t} \tilde{C}_{t} R_{t}^{-1} \dot{\mathbf{x}}_{t}+ R_{t} \tilde{J}_{t} R_{t}^{-1} \boldsymbol{\omega }_{t}+R_{t} \tilde{\mathbf{T}}^{\mathrm{sh}}_{t}\,, $$

where the matrices $K_{t}$, $C_{t}$, and $J_{t}$ are defined by

2.9 $$ \begin{aligned} \tilde{K}_{t}:=&\, \tilde{K}^{(1)}_{t}+\tilde{K}^{(2)}_{t}, \quad \text{with}\quad \tilde{K}^{(i)}_{t}:=[(C_{\parallel }-C_{\perp })\tilde{\mathbf{e}}^{(i)}_{t}\otimes \tilde{\mathbf{e}}^{(i)}_{t}+C_{\perp }I]\ell _{i} \,, \\ \tilde{C}_{t}:=&\, \tilde{C}^{(1)}_{t}+\tilde{C}^{(2)}_{t}, \quad \text{with}\quad \tilde{C}^{(i)}_{t}:=\frac{\ell _{i}^{2}}{2} C_{\perp }\tilde{E}^{(i)}_{t}\quad \text{and $\tilde{E}^{(i)}_{t}$ such that}\;\tilde{E}^{(i)}_{t} \mathbf{v}=\tilde{\mathbf{e}}^{(i)}_{t}\times \mathbf{v}, \\ \tilde{J}_{t}:=&\, \tilde{J}^{(1)}_{t}+\tilde{J}^{(2)}_{t}, \quad \text{with}\quad \tilde{J}^{(1)}_{t}:=\frac{\ell _{1}^{3}}{3}C_{\perp }[I-\tilde{\mathbf{e}}^{(1)}_{t} \otimes \tilde{\mathbf{e}}^{(1)}_{t}]+ \ell _{1}C_{\tau }\tilde{\mathbf{e}}^{(1)}_{t}\otimes \tilde{\mathbf{e}}^{(1)}_{t} \quad \text{and} \\ & \phantom{ \tilde{J}^{(1)}_{t}+\tilde{J}^{(2)}_{t},\quad \text{with}} \quad \tilde{J}^{(2)}_{t}:=\frac{\ell _{2}^{3}}{3}C_{\perp }[I- \tilde{\mathbf{e}}^{(2)}_{t}\otimes \tilde{\mathbf{e}}^{(2)}_{t}], \end{aligned} $$

and the viscous force and torque due to the shape deformation are

$$\tilde{\mathbf{F}}_{t}^{\mathrm{sh}}:=\frac{\ell _{2}^{2}}{2} C_{\perp }\dot{\tilde{\mathbf{e}}}_{t}^{(2)}\qquad \text{and}\qquad \tilde{\mathbf{T}}_{t}^{\mathrm{sh}}:=\frac{\ell _{2}^{3}}{3}C_{\perp }\tilde{E}^{(2)}_{t} \dot{\tilde{\mathbf{e}}}^{(2)}_{t}\,. $$

Expressions (2.7) and (2.8) can be written together in matricial form as

2.10 $$\begin{aligned} \left ( \textstyle\begin{array}{c} \mathbf{F}_{t} \\ \mathbf{T}_{t} \end{array}\displaystyle \right )=&\left [ \textstyle\begin{array}{c@{\quad }c} R_{t} &0 \\ 0 &R_{t} \end{array}\displaystyle \right ]\left [ \textstyle\begin{array}{c@{\quad }c} \tilde{K}_{t} &\tilde{C}_{t}^{\top }\\ \tilde{C}_{t} &\tilde{J}_{t} \end{array}\displaystyle \right ]\left [ \textstyle\begin{array}{c@{\quad }c} R_{t}^{-1} &0 \\ 0 &R_{t}^{-1} \end{array}\displaystyle \right ]\left ( \textstyle\begin{array}{c} \dot{\mathbf{x}}_{t} \\ \boldsymbol{\omega }_{t} \end{array}\displaystyle \right ) \\ &+\left [ \textstyle\begin{array}{c@{\quad }c} R_{t} &0 \\ 0 &R_{t} \end{array}\displaystyle \right ]\left ( \textstyle\begin{array}{c} \tilde{\mathbf{F}}^{\mathrm{sh}}_{t} \\ \tilde{\mathbf{T}}^{\mathrm{sh}}_{t} \end{array}\displaystyle \right ). \end{aligned}$$

The matrix

2.11 $$ \tilde{\mathcal{M}}_{t}:=\left [ \textstyle\begin{array}{c@{\quad }c} \tilde{K}_{t} &\tilde{C}_{t}^{\top }\\ \tilde{C}_{t} &\tilde{J}_{t} \end{array}\displaystyle \right ] $$

is known in the literature as the *grand resistance matrix*. It is a $6\times 6$ symmetric (see (2.9)) and positive-definite (see [23]) matrix.

Suppose that the two links are of equal lengths, $\ell _{1}=\ell _{2}=:L$. Listing also $\dot{\varphi }_{t}$ and $\dot{\vartheta }_{t}$ in the state of the system, and setting (2.10) equal to zero (this is sometimes called the *self-propulsion constraint*, see Remark 2.3), we have

2.12 $$ \left ( \textstyle\begin{array}{c} R_{t}^{-1}\dot{\mathbf{x}}_{t} \\ R_{t}^{-1}\boldsymbol{\omega }_{t} \\ \dot{\varphi }_{t} \\ \dot{\vartheta }_{t} \end{array}\displaystyle \right )= V_{1}(\varphi _{t},\vartheta _{t})u_{1}+V_{2}(\varphi _{t}, \vartheta _{t})u_{2}\,, $$

where

2.13 $$\begin{aligned} & V_{1}:=\left ( \textstyle\begin{array}{c} \tilde{\mathcal{M}}_{t}^{-1}\left ( \textstyle\begin{array}{c} -\frac{L^{2}}{2} C_{\perp }\cos \vartheta _{t}\cos \varphi _{t} \\ -\frac{L^{2}}{2} C_{\perp }\sin \vartheta _{t}\cos \varphi _{t} \\ \frac{L^{2}}{2} C_{\perp }\sin \varphi _{t} \\ \frac{L^{3}}{3} C_{\perp }\sin \vartheta _{t} \\ -\frac{L^{3}}{3} C_{\perp }\cos \vartheta _{t} \\ 0 \end{array}\displaystyle \right ) \\ 1 \\ 0 \end{array}\displaystyle \right ), \\ &V_{2}:=\left ( \textstyle\begin{array}{c} \tilde{\mathcal{M}}_{t}^{-1}\left ( \textstyle\begin{array}{c} \frac{L^{2}}{2} C_{\perp }\sin \vartheta _{t}\sin \varphi _{t} \\ -\frac{L^{2}}{2} C_{\perp }\cos \vartheta _{t}\sin \varphi _{t} \\ 0 \\ \frac{L^{3}}{6} C_{\perp }\cos \vartheta _{t}\sin 2\varphi _{t} \\ \frac{L^{3}}{6} C_{\perp }\sin \vartheta _{t}\sin 2\varphi _{t} \\ -\frac{L^{3}}{3} C_{\perp }\sin ^{2}\varphi _{t} \end{array}\displaystyle \right ) \\ 0 \\ 1 \end{array}\displaystyle \right ) \end{aligned}$$

and $u_{1},u_{2}:[0,T]\to R$ are measurable functions. By straightforward computations we have

2.14 $$ V_{1}=\left ( \textstyle\begin{array}{c} \displaystyle \frac{LC_{\perp }\cos \vartheta _{t}\sin ^{2}\frac{\varphi _{t}}{2}}{2(C_{\perp }+C_{\parallel }+(C_{\parallel }-C_{\perp })\cos \varphi _{t})} \\ \displaystyle \frac{LC_{\perp }\sin \vartheta _{t}\sin ^{2}\frac{\varphi _{t}}{2}}{2(C_{\perp }+C_{\parallel }+(C_{\parallel }-C_{\perp })\cos \varphi _{t})} \\ \displaystyle \frac{LC_{\perp }\sin \varphi _{t}}{4(C_{\perp }+C_{\parallel }+(C_{\parallel }-C_{\perp })\cos \varphi _{t})} \\ \displaystyle \frac{\sin \vartheta _{t}}{2} \\ \displaystyle -\frac{\cos \vartheta _{t}}{2} \\ 0 \\ 1 \\ 0 \end{array}\displaystyle \right )\quad \text{and} $$

2.15 $$ V_{2}=\left ( \textstyle\begin{array}{c} \displaystyle \frac{-24 C_{\tau }L \sin \vartheta _{t} \sin ^{2}\frac{\varphi _{t} }{2} \sin \varphi _{t}}{36 C_{\tau }\cos \varphi _{t} -45 C_{\tau }+\cos 2 \varphi _{t} \left (2 C_{\perp }L^{2}-15 C_{\tau }\right )-2 C_{\perp }L^{2}} \\ \displaystyle \frac{24 C_{\tau }L \cos \vartheta _{t} \sin ^{2}\frac{\varphi _{t} }{2} \sin \varphi _{t}}{36 C_{\tau }\cos \varphi _{t} -45 C_{\tau }+\cos 2 \varphi _{t} \left (2 C_{\perp }L^{2}-15 C_{\tau }\right )-2 C_{\perp }L^{2}} \\ 0 \\ \displaystyle \frac{-3 C_{\tau }\cos \vartheta _{t} (5 \sin 2 \varphi _{t} -6 \sin \varphi _{t} )}{36 C_{\tau }\cos \varphi _{t} -45 C_{\tau }+\cos 2 \varphi _{t} \left (2 C_{\perp }L^{2}-15 C_{\tau }\right )-2 C_{\perp }L^{2}} \\ \displaystyle \frac{-3 C_{\tau }\sin \vartheta _{t} (5 \sin 2 \varphi _{t} -6 \sin \varphi _{t} )}{36 C_{\tau }\cos \varphi _{t} -45 C_{\tau }+\cos 2 \varphi _{t} \left (2 C_{\perp }L^{2}-15 C_{\tau }\right )-2 C_{\perp }L^{2}} \\ \displaystyle \frac{4L^{2}C_{\perp }\sin ^{2}\varphi _{t}}{36 C_{\tau }\cos \varphi _{t} -45 C_{\tau }+\cos 2 \varphi _{t} \left (2 C_{\perp }L^{2}-15 C_{\tau }\right )-2 C_{\perp }L^{2}} \\ 0 \\ 1 \end{array}\displaystyle \right ). $$

### Remark 2.1

The vector fields $V_{1}$ and $V_{2}$ are analytic since the denominators in the expressions for their components are always nonzero for positive values of $C_{\perp}$, $C_{\parallel}$, $C_{\tau}$, and $L$. This is straightforward to verify for $V_{1}$. For $V_{2}$, the denominators are zero if and only if

2.16 $$ \left (\frac{2 C_{\perp}L^{2}}{C_{\tau}} - 15\right )\chi ^{2} + 18\chi - \left (\frac{2 C_{\perp}L^{2}}{C_{\tau}} + 15\right ) = 0, $$

where $\chi = \cos \varphi _{t}$. For $C_{\perp}L^{2}/C_{\tau}> 0$, the quadratic equation (2.16) has no roots $\chi $ in the interval $[-1,1]$, hence, the denominators in $V_{2}$ are always nonzero.

The following theorem, whose proof can be obtained by applying classical results from ODE theory (see, *e.g.*, [21]), holds. Notice that the controllability Theorem 3.6 below provides a sufficient condition for existence.

### Theorem 2.2

*Let* $\left(\bar{x},\bar{R},\bar{\varphi },\bar{\vartheta })\in R^{3}\times SO(3)\times (0,\pi )\times (-\pi ,\pi \right)$ *be given*. *There exists a unique absolutely continuous solution* $\left(x_{t},R_{t},\varphi_{t},\vartheta_{t}):[0,+\infty )\to R^{3}\times SO(3)\times (0,\pi )\times (-\pi ,\pi \right)$ *to the Cauchy problem for* (2.12) *with initial condition* $(\mathbf{x}_{0},R_{0},\varphi _{0},\vartheta _{0})=( \bar{\mathbf{x}},\bar{R},\bar{\varphi},\bar{\vartheta})$, *for any controls* $u_{1},u_{2}\in L^{\infty }(0,+\infty )$.

### Remark 2.3

We comment here briefly on the modeling assumptions of our system, namely the use of Resistive Force Theory, the self-propulsion constraint, and the special treatment of link 1 regarding rotational drag.

- Resistive Force Theory was introduced in [19] and prescribes that the viscous force and torque per unit length acting on a slender body immersed in a low Reynolds number fluid are linear with respect to the local tangential and normal components of the velocity of the body (see formulas (2.4a)–(2.4c)). This approximation is meaningful for filament-like bodies and is popular in the community of biological fluid dynamicists, as it provides a simple and concise way to compute these forces and torques in flagellated microorganisms, see, *e.g.*, [16, 24, 34]. The same approach is also used to model swimming microrobots, see, *e.g.*, [33, 41].
- The self-propulsion constraint, namely setting the total viscous force and torque $(\mathbf{F}_{t},\mathbf{T}_{t})$ from (2.10) equal to zero, is enforced to exclude the presence of any intervention from the external environment. This is the physical meaning of the mathematical relationship $(\mathbf{F}_{t},\mathbf{T}_{t})=(\boldsymbol{0},\boldsymbol{0})$ for all $t\in [0,+\infty )$. At a practical level, in view of the applications to robotics, this means that the swimmer propels itself solely with internally actuated shape changes rather than external effects.
- The constraints $0< C_{\parallel }$, $C_{\perp }<+\infty $ on the drag coefficients reflect the fact that drag forces oppose the direction of motion; for a slender body, it is verified that $C_{\parallel}< C_{\perp}$ which, instead, reflects the fact it is easier to move along the tangential direction than sideways. The ratio $C_{\perp }/C_{\parallel }\approx 2$ is obtained for cylinders of high aspect ratio in Stokes flow [19, 22]. The expressions in (2.4a)–(2.4c) and the self-propulsion constraint can be interpreted as constraints of non-holonomic type. In the limit $C_{\perp }/C_{\parallel }\to +\infty $, it is possible to recover the dynamics on an anisotropic frictional environment, see, *e.g.*, [7, 8, 11, 37].
- The final term in (2.4b) represents the viscous torque due to rotations of a cylinder about its axis. A corresponding term for the torque density on link 2 could be included in (2.4c) but for slender rods, it is generally expected to be smaller in magnitude than the torque due to motion of the link perpendicular to its axis [28]. We have verified that including the torsional drag term on link 2 does not alter analyticity of the vector fields. In this case, controllability of the 2-link swimmer can be proved in the same way as in Theorem 3.6 below, for almost every $(C_{\parallel},C_{\perp},C_{\tau},L)\in (0,+\infty )^{4}$. From the modeling point of view, including $C_{\tau}$ on only the first link corresponds to the first link being a thicker cylinder than the second, providing a minimal description of swimmers (or microrobots) that have a large head or body and a thin flagellum [6, 36].

## Controllability

### Preliminaries

In this subsection we present the basic notions about control systems on Lie groups. We use their properties in order to state the controllability results for the 2-link swimmer in Sect. 3.2.

Let $G$ be an $n$-dimensional matrix Lie group and let $\mathcal{S}$ be an $m$-dimensional manifold; we call $\mathbf{M}:=G\times \mathcal{S}$ the *configuration space*, whose generic element is $\mathbf{z}:=(g,s)$.

#### Definition 3.1

A *nonlinear control system on* $G$ is an ODE of the form

3.1 $$ \dot{\mathbf{z}}= \begin{pmatrix} \dot{g} \\ \dot{s} \end{pmatrix} = \begin{pmatrix} g \xi (s,u) \\ u \end{pmatrix} , $$

where $\xi $ is a map from the tangent space $T\mathcal{S}$ to the Lie algebra $\mathfrak{g}$ of $G$ which is linear in the fibers, i.e.,

$$ \xi (s,u)=\sum _{i=1}^{m} \xi _{i}(s)u_{i},\qquad \mbox{for some analytic (nonlinear) maps}\ \xi _{i}\colon \mathcal{S}\to \mathfrak{g},\quad i=1,\ldots ,m, $$

and $u:[0,T]\to (u_{1}(t),\ldots ,u_{m}(t))\in T_{s}S≃R^{m}$ is the vector of controls.

Denoting by ${\hat{e}}_{i}^{R^{m}}$ the elements of the canonical basis of $R^{m}$, system (3.1) can be written as

3.2 $$\dot{z}=\sum_{i=1}^{m}\left(\begin{array}{c} g\xi_{i}(s)\\ {\hat{e}}_{i}^{R^{m}} \end{array}\right)u_{i}=:\sum_{i=1}^{m}Z_{i}(g,s)u_{i},$$

where $Z_{i}=(Z_{i}^{G},Z_{i}^{S}):S\to T_{g}G\times T_{s}S≃T_{g}G\times R^{m}$, for $i\in \{1,\ldots ,m\}$.

#### Definition 3.2

Let $g\in G$. A vector field $X$ on $\mathbf{M}$ is *equivariant* with respect to the group action

3.3 $$ \Psi _{g}\colon \mathbf{M}\to \mathbf{M}, \qquad \mathbf{z}=(h,s) \mapsto \Psi _{g}(\mathbf{z}):=(g h,s) $$

if, denoting by $(\cdot )_{*}$ the push-forward,

3.4 $$ (\Psi _{g})_{*} X(\mathbf{z})=X(\mathbf{z}), \qquad \text{for $\mathbf{z}=(h,s)\in \mathbf{M}$}. $$

By the definition of push-forward, the left-hand side in (3.4) is $\big ((D\Psi _{g})(\Psi _{g}^{-1}(\mathbf{z}))\big )\cdot X(\Psi _{g}^{-1}( \mathbf{z}))$, where $D$ denotes the differential; since $\Psi _{g}$ defined in (3.3) is nothing but the left-translation by $g$ in the $G$-component of $\mathbf{z}$, it turns out that

$$\big((D\Psi _{g})(\Psi _{g}^{-1}(\mathbf{z}))\big)= \begin{pmatrix} T_{e} L_{g} & 0 \\ 0 & I_{m} \end{pmatrix} = \begin{pmatrix} g & 0 \\ 0 & I_{m} \end{pmatrix} , $$

where $L_{g}$ is the left translation by $g\in G$ (namely, $L_{g} h=gh$), $T_{e}$ is the tangent map to the identity $e\in G$, and $I_{m}$ is the $m$-dimensional identity matrix.

#### Remark 3.3

The following observations are straightforward:

(i) for any $\bar{g}\in G$, the vector fields $Z_{i}$ ($i=1, \ldots ,m$) in (3.2) are equivariant with respect to the group action $\Psi _{\bar{g}}$ defined in (3.3);
(ii) for any $Z_{i},Z_{j}\in T_{g}G\times R^{m}$ and for any $\bar{g}\in G$, the Lie bracket $[Z_{i},Z_{j}]$ is equivariant with respect to the group action $\Psi _{\bar{g}}$.

We now give the definition of controllability.

#### Definition 3.4

The nonlinear control system (3.1) is said to be *controllable* if for any initial $(\bar{g}^{0},\bar{s}^{0})\in \mathbf{M}$ and final $(\bar{g}^{1},\bar{s}^{1})\in \mathbf{M}$ there exist a time $T>0$ and a measurable and bounded control $u:[0,T]\to R^{m}$ such that $(g_{0},s_{0})=(\bar{g}^{0},\bar{s}^{0})$ and $(g_{T},s_{T})=(\bar{g}^{1},\bar{s}^{1})$ where $(g_{t},s_{t})\colon [0,T]\to \mathbf{M}$ is the unique solution to (3.1) with control $u$.

The following statement of the Orbit Theorem can be easily derived from [25, Chap. 2, Theorems 1 and 2].

#### Theorem 3.5

The orbit theorem

*Let* $\mathbf{M}$ *be an analytic manifold*, *and let* $\mathcal{Z}$ *be a family of analytic vector fields on* $\mathbf{M}$. *Then*

- *each orbit of* $\mathcal{Z}$ *is an analytic submanifold of* $\mathbf{M}$, *and*
- *if* $\mathbf{N}$ *is an orbit of* $\mathcal{Z}$, *then the tangent space of* $\mathbf{N}$ *at* $\mathbf{z}$ *is given by* $\mathfrak{Lie}_{\mathbf{z}}(\mathcal{Z})$. *In particular*, *the dimension of* $\mathfrak{Lie}_{\mathbf{z}}(\mathcal{Z})$ *is constant as* $\mathbf{z}$ *varies on* $\mathbf{N}$.

### The Controllability Theorem

We are interested in studying how the shape change of our swimmer determines its spatial position and orientation in the framework of control systems on Lie groups. We will work with $\mathbf{M}=G\times \mathcal{S}=SE(3)\times (0,\pi )\times (-\pi ,\pi )$, by posing

3.5 $$ g:=\begin{pmatrix} R(\alpha ,\beta ,\gamma ) & \tau \\ 0 & 1 \end{pmatrix} \in SE(3)\quad \text{and $s:=(\varphi ,\vartheta )\in (0,\pi )\times (-\pi ,\pi )$,} $$

where $R(\alpha ,\beta ,\gamma )\in SO(3)$ (with $\alpha $, $\beta $, and $\gamma $ the Euler angles) and $\tau :={\left(x_{1},x_{2},x_{3}\right)}^{⊤}\in R^{3}$. In order to write system (3.2) in vector form, we introduce the Lie algebra isomorphism $L:R^{6}\to \mathrm{se}(3)$ defined by

$$ y=(y_{1},\ldots ,y_{6})^{\top }\mapsto \begin{pmatrix} 0 & -y_{6} & y_{5} & y_{1} \\ y_{6} & 0 & -y_{4} & y_{2} \\ -y_{5} & y_{4} & 0 & y_{3} \\ 0 & 0 & 0 & 0 \end{pmatrix} . $$

The application of $\mathcal{L}^{-1}$ to the $g$-component in (3.2) will transform it from a $4\times 4$-matrix into a vector in $R^{6}$. Moreover, denoting by $Z^{G}$ and $Z^{\mathcal{S}}$ the $G$- and $\mathcal{S}$-components, respectively, of any $Z\in T_{g}SE(3)\times R^{2}$, Remark 3.3(ii) implies that, for any $Z_{1},Z_{2}\in T_{g}SE(3)\times R^{2}$,

3.6 $${\left(\Psi_{g}^{-1}\right)}_{*}{\left[Z_{1},Z_{2}\right]}_{T_{g}SE(3)\times R^{2}}={\left[{\left(\Psi_{g}^{-1}\right)}_{*}Z_{1},{\left(\Psi_{g}^{-1}\right)}_{*}Z_{2}\right]}_{\mathrm{se}(3)\times R^{2}}.$$

Moreover, since ℒ is a Lie algebra isomorphism, if $Z_{i}=(g\xi _{i}(s),\mathbf{a}_{i})$, $a_{i}\in R^{2}$, $i=1,2$, we can rewrite (3.6) as

$$\begin{array}{rl} \left(\begin{array}{c} L^{-1}\Gamma^{G}\\ \Gamma^{S} \end{array}\right)= & {\left[\left(\begin{array}{c} L^{-1}{\left({\left(\Psi_{g}^{-1}\right)}_{*}Z_{1}\right)}^{G}\\ {\left({\left(\Psi_{g}^{-1}\right)}_{*}Z_{1}\right)}^{S} \end{array}\right),\left(\begin{array}{c} L^{-1}{\left({\left(\Psi_{g}^{-1}\right)}_{*}Z_{2}\right)}^{G}\\ {\left({\left(\Psi_{g}^{-1}\right)}_{*}Z_{2}\right)}^{S} \end{array}\right)\right]}_{R^{8}}\\ = & \left(\begin{array}{c} L^{-1}({\left[\xi_{1},\xi_{2}\right]}_{\mathrm{se}(3)}+(\nabla_{s}\xi_{2})a_{1}-(\nabla_{s}\xi_{1})a_{2})\\ 0_{2} \end{array}\right), \end{array}$$

where we have denoted by $\Gamma $ the left-hand side in (3.6), and where $\boldsymbol{0}_{2}$ denotes the zero vector in $R^{2}$. We recall here that $[\xi _{1},\xi _{2}]_{\mathfrak{se}(3)}=\xi _{1}\xi _{2}-\xi _{2}\xi _{1}$ is the commutator, for any $\xi _{1},\xi _{2}\in \mathfrak{se}(3)$.

We can now state the controllability theorem for the 2-link swimmer.

#### Theorem 3.6

Controllability of the 2-link

*The* 2-*link swimmer is controllable in the sense of Definition *3.4*if* $C_{\perp}\neq C_{\parallel}$.

#### Proof

The proof is divided into three steps.

*Step 1.* By (3.5), the equations of motion (2.12) can be cast in the form

3.7 $$\left(\begin{array}{c} L^{-1}(g^{-1}\dot{g})\\ \dot{s} \end{array}\right)=V_{1}(s)u_{1}+V_{2}(s)u_{2}=:\left(\begin{array}{c} L^{-1}\xi_{1}(s)\\ {\hat{e}}_{1}^{R^{2}} \end{array}\right)u_{1}+\left(\begin{array}{c} L^{-1}\xi_{2}(s)\\ {\hat{e}}_{2}^{R^{2}} \end{array}\right)u_{2}.$$

In (3.7), we notice that $g^{-1}\dot{g}\in \mathfrak{se}(3)$; the action of $g^{-1}$ on an element $\dot{g}$ of the tangent space $T_{g} SE(3)$ can be written as

$$ \begin{pmatrix} R^{-1}(\alpha ,\beta ,\gamma ) & -\tau \\ 0 & 1 \end{pmatrix} \dot{g};$$

$V_{1}(s)$, $V_{2}(s)$, $\mathcal{L}^{-1}\xi _{1}(s)$, $\mathcal{L}^{-1}\xi _{2}(s)$ can be found in (2.13), (2.14), (2.15). Finally, $u_{1},u_{2}:[0,T]\to R$ are the *control functions*. It is a well-known fact that if $u_{1}$, $u_{2}$ are taken in $L^{\infty }(0,T)$, there exists a unique absolutely continuous solution to (3.7) [26, Lemma 2.1].

We now remark that, since ℒ is an isomorphism, system (3.7) is exactly a control system on the Lie group $SE(3)$ according to Definition 3.1, and thus the control vector fields are equivariant with respect to the $SE(3)$ action, as pointed out in Remark 3.3(i).

*Step 2.* By Remark 3.3(ii) and the Rashevsky–Chow Theorem (see [1, Theorem 5.9]), to prove the controllability of the system at a point $(h,s^{*})$ it suffices to compute the Lie brackets of the vector fields $V_{i}$ at the point $(e,s^{*})$ and to show that they generate any directions in the Lie algebra $\mathfrak{se}(3)$. Let

$$\begin{aligned} V_{3} &:=[V_{1},V_{2}],\quad V_{4}:=[V_{1},V_{3}],\quad V_{5}:=[V_{2},V_{3}],\\ V_{6}&:=[V_{1},V_{5}],\quad V_{7}:=[V_{2},V_{5}],\quad V_{8}:=[V_{3},V_{4}]. \end{aligned}$$

A simple computation of these Lie brackets at the point $(e,s^{*})=\big (e,(\varphi ^{*},\vartheta ^{*})\big )=\big (e,( \frac{\pi }{2},0)\big )$ yields

$$V_{1}^{*}=V_{1}(s^{*})=\left ( \textstyle\begin{array}{c} \displaystyle \frac{LC_{\perp}}{4(C_{\perp}+C_{\parallel})}\\ 0\\ \displaystyle \frac{LC_{\perp}}{4(C_{\perp}+C_{\parallel})}\\ 0\\ \displaystyle -\frac{1}{2}\\ 0\\ 1\\ 0 \end{array}\displaystyle \right ),\qquad V_{2}^{*}=V_{2}(s^{*})=\left ( \textstyle\begin{array}{c} 0\\ \displaystyle -\frac{6 C_{\tau}L}{15 C_{\tau}+2 L^{2}C_{\perp}}\\ 0\\ \displaystyle -\frac{9C_{\tau}}{15 C_{\tau}+2 L^{2}C_{\perp}}\\ 0\\ \displaystyle -\frac{2L^{2}C_{\perp}}{15 C_{\tau}+2 L^{2}C_{\perp}}\\ 0\\ 1 \end{array}\displaystyle \right ), $$

$$ V_{3}^{*}:=V_{3}(s^{*})= \left ( \textstyle\begin{array}{c} 0\\ \displaystyle -\frac{6 C_{\tau}L \left (3 C_{\tau}(4C_{\perp}-C_{\parallel}) +2 C_{\perp}L^{2}(2C_{\perp}+C_{\parallel})\right )}{(15 C_{\tau}+2 L^{2}C_{\perp})^{2}(C_{\perp}+C_{\parallel})}\\ 0\\ \displaystyle -\frac{3C_{\tau}(234 C_{\tau}+60 C_{\perp}L^{2})}{4(15 C_{\tau}+2 L^{2}C_{\perp})^{2}}\\ 0\\ \displaystyle \frac{27C_{\tau}(2 C_{\perp}L^{2}-5C_{\tau})}{2(15 C_{\tau}+2 L^{2}C_{\perp})^{2}}\\ 0\\ 0 \end{array}\displaystyle \right ), $$

$$ \begin{aligned} &V_{4}^{*}:=V_{4}(s^{*})=\\ & \left ( \textstyle\begin{array}{c} 0\\ \frac{3 L C_{\tau}\left (12L^{2}C_{\tau}C_{\perp}(9C_{\perp}^{2}+11C_{\perp}C_{\parallel}+22 C_{\parallel})-4L^{4} C_{\perp}^{2}(7C_{\perp}^{2}+13C_{\perp}C_{\parallel}+2C_{\parallel}^{2})+9C_{\tau}^{2}(121 C_{\perp}^{2}+147C_{\perp}C_{\parallel}+126C_{\parallel}^{2})\right )}{(15 C_{\tau}+2 L^{2}C_{\perp})^{3}(C_{\perp}+C_{\parallel})^{2}}\\ 0\\ \frac{9 C_{\tau}\left (3933C_{\tau}^{2}+540L^{2}C_{\tau}C_{\perp}-44L^{4} C_{\perp}^{2}\right )}{4(15 C_{\tau}+2 L^{2}C_{\perp})^{3}}\\ 0\\ -\frac{3C_{\tau}(2385C_{\tau}^{2}+396L^{2} C_{\tau}C_{\perp}-220L^{4} C_{\perp}^{2})}{4(15 C_{\tau}+2 L^{2}C_{\perp})^{3}}\\ 0\\ 0 \end{array}\displaystyle \right ), \end{aligned} $$

$$V_{5}^{*}:=V_{5}(s^{*})= \left ( \textstyle\begin{array}{c} \displaystyle \frac{9 LC_{\tau}^{2} (11 C_{\perp}+ C_{\parallel})}{(15 C_{\tau}+2 L^{2}C_{\perp})^{2} (C_{\perp}+C_{\parallel})}\\ 0\\ \displaystyle -\frac{27L C_{\tau}^{2}(C_{\perp}+3C_{\parallel})}{(15 C_{\tau}+2 L^{2}C_{\perp})^{2}(C_{\perp}+C_{\parallel})}\\ 0\\ \displaystyle -\frac{216 C_{\tau}^{2}}{(15 C_{\tau}+2 L^{2}C_{\perp})^{2}}\\ 0\\ 0\\ 0 \end{array}\displaystyle \right ), $$

$$\begin{aligned} &V_{6}^{*}:=V_{6}(s^{*})\\ &= \left ( \textstyle\begin{array}{c} \frac{9LC_{\tau}^{2}\left (10L^{2}C_{\perp}(C_{\perp}-C_{\parallel})(3C_{\perp}+7C_{\parallel})-3C_{\tau}(189C_{\perp}^{2}+188C_{\perp}C_{\parallel}+199C_{\parallel}^{2})\right )}{2(15 C_{\tau}+2 L^{2}C_{\perp})^{3}(C_{\perp}+C_{\parallel})^{2}} \\ 0\\ \frac{27L C_{\tau}\left (2L^{2}C_{\perp}(C_{\perp}-C_{\parallel})(3C_{\perp}+7C_{\parallel})+3C_{\tau}(39C_{\perp}^{2}+116C_{\perp}C_{\parallel}+37C_{\parallel}^{2})\right )}{2(15 C_{\tau}+2 L^{2}C_{\perp})^{3}(C_{\perp}+C_{\parallel})^{2}} \\ 0\\ \frac{7776C_{\tau}^{3}}{(15 C_{\tau}+2 L^{2}C_{\perp})^{3}}\\ 0\\ 0\\ 0 \end{array}\displaystyle \right ), \end{aligned}$$

$$V_{7}^{*}:=V_{7}(s^{*})= \left ( \textstyle\begin{array}{c} 0\\ \displaystyle \frac{54LC_{\tau}^{3}(23C_{\perp}-11C_{\parallel})}{(15 C_{\tau}+2 L^{2}C_{\perp})^{3}(C_{\perp}+C_{\parallel})}\\ 0\\ \displaystyle \frac{3240C_{\tau}^{3}}{(15 C_{\tau}+2 L^{2}C_{\perp})^{3}}\\ 0\\ \displaystyle \frac{1944C_{\tau}^{3}}{(15 C_{\tau}+2 L^{2}C_{\perp})^{3}}\\ 0\\ 0 \end{array}\displaystyle \right ), $$

$$ \begin{aligned} &V_{8}^{*}:=V_{8}(s^{*}) \\ &= \left ( \textstyle\begin{array}{c} -\frac{9LC_{\tau}^{2}\big(-15C_{\tau}(23C_{\perp}^{2}+24 C_{\perp}C_{\parallel}+13C_{\parallel}^{2})+2L^{2}C_{\perp}(47C_{\perp}^{2}+48C_{\perp}C_{\parallel}+37 C_{\parallel}^{2})\big)}{(15 C_{\tau}+2 L^{2}C_{\perp})^{3}(C_{\perp}+C_{\parallel})^{2}}\\ 0\\ \frac{27LC_{\tau}^{2}\big(3C_{\tau}(7C_{\perp}^{2}-24 C_{\perp}C_{\parallel}-83 C_{\parallel}^{2})+2L^{2}C_{\perp}(13C_{\perp}^{2}+32C_{\perp}C_{\parallel}-C_{\parallel}^{2})\big)}{2(15 C_{\tau}+2 L^{2}C_{\perp})^{2}(C_{\perp}+C_{\parallel})^{2}}\\ 0\\ \frac{54C_{\tau}^{2}(22L^{2}C_{\perp}-75C_{\tau})}{(15 C_{\tau}+2 L^{2}C_{\perp})^{3}}\\ 0\\ 0\\ 0 \end{array}\displaystyle \right ). \end{aligned} $$

The computation of the determinant of the $8\times 8$ matrix $(V_{1}^{*}|\cdots |V_{8}^{*})$ gives

3.8 $$ \delta ^{*}:=\det (V_{1}^{*}|\cdots |V_{8}^{*})_{( \frac{\pi }{2},0)}= \frac{p(C_{\parallel }, C_{\perp }, C_{\tau }, L)}{q(C_{\parallel }, C_{\perp }, C_{\tau }, L)},$$

where $p$ and $q$ are polynomials whose explicit expressions are

3.9 $$ \begin{aligned} p=&\, 2,\!754,\!990, \!144\, L^{3}C_{\tau}^{11}(C_{\perp}-C_{\parallel})^{3}(3C_{\perp}+7C_{\parallel})^{3}(7C_{\tau}+10L^{2}C_{\perp})\\ q=&\, (15 C_{\tau}+2 L^{2}C_{\perp})^{12}(C_{\perp}+C_{\parallel})^{6}. \end{aligned} $$

Notice that $\delta ^{*}\neq 0$ if $C_{\perp}\neq C_{\parallel}$. This proves that, through the iterated Lie brackets, it is possible to generate the 8-dimensional tangent space $\mathrm{se}(3)\times R^{2}$ at the point $(h,s^{*})=(e,(\frac{\pi }{2},0))$ for every $(C_{\parallel }, C_{\perp }, C_{\tau }, L)\in (0,+\infty )^{4}$ with $C_{\perp }\neq C_{\parallel }$. Controllability at $(h,s^{*})$ follows.

*Step 3.* In Step 2, we proved that $\dim \big (\mathfrak{Lie}(\{V_{1},V_{2}\})_{(e,(\frac{\pi }{2},0))}\big )=8$. Let us now consider an arbitrary point $s\in (0,\pi )\times (-\pi ,\pi )$. Since the shape variable can be steered directly by means of the control functions $u_{i}$, the shape configuration $s$ can be reached from the shape configuration $s^{*}=(\frac{\pi }{2},0)$. This shape change results in the group component changing from $h=e$ to a certain $h'\in SE(3)$, so that the point $(h',s)$ belongs to the orbit of the point $(h,s^{*})=(e,(\frac{\pi }{2},0))$. Since the vector fields $V_{i}$ are analytic, by the Orbit Theorem 3.5 the dimension of the Lie algebra is preserved along the orbit, so that

$$ \dim \big(\mathfrak{Lie}(\{V_{1},V_{2}\})_{(h',s)}\big)=\dim \big( \mathfrak{Lie}(\{V_{1},V_{2}\})_{(e,(\frac{\pi }{2},0))}\big)=8.$$

Finally, thanks to the equivariance of the vector fields with respect to the group action (see Remark 3.3(ii) and (3.6)), we have that

$$ \dim \big(\mathfrak{Lie}(\{V_{1},V_{2}\})_{(e,s)}\big)=\dim \big( \mathfrak{Lie}(\{V_{1},V_{2}\})_{(h',s)}\big)=8.$$

Thus, $V_{1}$ and $V_{2}$ generate the tangent space $\mathrm{se}(3)\times R^{2}$ at $(e,s)$. By the arbitrariness of $s$, controllability follows from the Rashevsky–Chow Theorem. □

#### Remark 3.7

The controllability result proved in Theorem 3.6 for the shape space given by $\mathcal{S}=(0,\pi )\times (-\pi ,\pi )$ can be generalized to include all of the points in $S^{2}$. Indeed, starting from $s^{*}=(\frac{\pi }{2},0)$, if we want to reach a point $\bar{s}\in \partial \big ((0,\pi )\times (-\pi ,\pi )\big )$ it is enough to choose the appropriate parametrization of the sphere which contains both $s^{*}$ and $\bar{s}$ (see [14, Example 1, page 55]).

#### Remark 3.8

By standard results on control theory [12, 40], controllability is ensured with *bounded* controls, thus for any final time $T<+\infty $ the 2-link swimmer is controllable by means of absolutely continuous shape parameters $(\varphi _{t},\vartheta _{t})\in (0,\pi )\times (-\pi ,\pi )$ for all $t\in [0,T]$ (see the $\mathcal{S}$-component of (3.7)).

### The Planar Scallop Theorem

We briefly discuss the well known Scallop Theorem [35] in the context of Theorem 3.6. If a force- and torque-free body in Stokes flow performs a sequence of shape changes that returns to the initial shape and is identical when the sequence is reversed in time, then the body will return to the initial position and orientation. The speed of progression through the pattern of shape changes does not affect the position at any given point along the sequence. This behavior is a consequence of the linearity and absence of explicit time dependence in the equations of Stokes flow.

A “scallop” consisting of two links connected by a hinge has a single, angular, degree of freedom. No periodic sequence of opening and closing of the hinge will result in a net displacement per cycle since each opening motion will be reversed by a closing motion. Controllability of our 2-link swimmer requires the second degree of freedom, $\vartheta _{t}$, and the torsional resistance coefficient $C_{\tau }> 0$, as the following proposition demonstrates.

#### Proposition 3.9

*If* $C_{\tau }=0$, *the* 2-*link swimmer is not controllable*, *so that the well*-*known Scallop Theorem is recovered*.

#### Proof

Setting $C_{\tau }=0$ in (2.14) and (2.15) we have that

3.10 $$ V_{1}=\left ( \textstyle\begin{array}{c} \displaystyle \frac{LC_{\perp }\cos \vartheta _{t}\sin ^{2}\frac{\varphi _{t}}{2}}{2(C_{\perp }+C_{\parallel }+(C_{\parallel }-C_{\perp })\cos \varphi _{t})} \\ \displaystyle \frac{LC_{\perp }\sin \vartheta _{t}\sin ^{2}\frac{\varphi _{t}}{2}}{2(C_{\perp }+C_{\parallel }+(C_{\parallel }-C_{\perp })\cos \varphi _{t})} \\ \displaystyle \frac{LC_{\perp }\sin \varphi _{t}}{4(C_{\perp }+C_{\parallel }+(C_{\parallel }-C_{\perp })\cos \varphi _{t})} \\ \displaystyle \frac{\sin \vartheta _{t}}{2} \\ \displaystyle -\frac{\cos \vartheta _{t}}{2} \\ 0 \\ 1 \\ 0 \end{array}\displaystyle \right )\quad \text{and}\quad V_{2}=\left ( \textstyle\begin{array}{c} 0 \\ 0 \\ 0 \\ 0 \\ 0 \\ -1 \\ 0 \\ 1 \end{array}\displaystyle \right ); $$

moreover all of the higher-order brackets $V_{3},\ldots ,V_{8}$ introduced in the proof of Theorem 3.6 vanish, so that $\det (V_{1}|\cdots |V_{8})\equiv 0$ and the vector fields $V_{1},\ldots ,V_{8}$ cannot generate the tangent space $\mathrm{se}(3)\times R^{2}$. Also notice that the expression of $V_{2}$ yields that $-\dot{\vartheta}$ coincides with one direction of the Lie algebra. The physical interpretation of this is that whenever we move the shape angle $\vartheta $, the system reacts with a counter-rotation of the body frame by the same angle, so that the 2-link swimmer does not leave the plane determined by the two links at the initial time. In this case, the angle $\vartheta $ cannot be considered as a proper shape parameter. Therefore, for $C_{\tau }=0$, the system has only one shape parameter which makes it equivalent to a planar scallop (see [35]). In particular, the only control vector field is $V_{1}$, which alone cannot generate the Lie algebra. □

## The $N$-Link Swimmer

In this section, we extend the results obtained in the previous sections to the $N$-link swimmer. We consider a slender swimmer composed of a chain of $N>2$ links of length $\ell _{i}\geqslant 0$ hinged at their extremities and moving in an infinite viscous fluid. In order to avoid degeneracy, we require that there exist at least $i,j\in \{1,\ldots ,N\}$, $i\neq j$, such that $\ell _{i}>0$ and $\ell _{j}>0$.

To provide a dynamical description of the $N$-link swimmer, we follow the construction of Sect. 2: each link is described by two angles $(\varphi _{t}^{(i)},\vartheta _{t}^{(i)})\in (0,\pi )\times (-\pi , \pi )$ that identify the direction of the link with respect to the co-moving frame. The angles $\{\varphi ^{(i)},\vartheta ^{(i)}\}_{i=2}^{N}$ are the shape parameters of the system and we will prove that the swimmer is able to move in the fluid once the time evolution of the $2N-2$ functions $t \mapsto \varphi _{t}^{(i)}$ and $t \mapsto \vartheta _{t}^{(i)}$ are given.

**Fig. 2:**
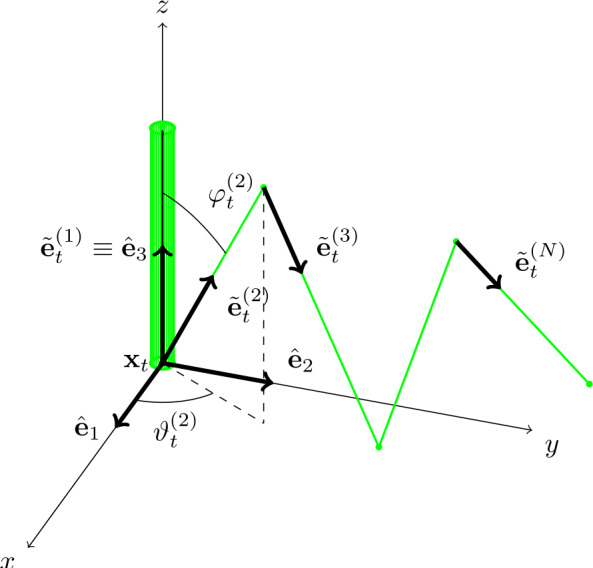
In black, the co-moving frame of the $N$-link swimmer; in green, the swimmer itself, with the thicker link 1 aligned with the $z$ axis. (Color figure online)

The unit vectors that describe the directions of the links are (see Fig. 2)

$$ \tilde{\mathbf{e}}_{t}^{(1)} :=\hat{\mathbf{e}}_{3} \, , \qquad \tilde{\mathbf{e}}_{t}^{(i)} :=\begin{pmatrix} \cos \vartheta _{t}^{(i)} \sin \varphi _{t}^{(i)} \\ \sin \vartheta _{t}^{(i)} \sin \varphi _{t}^{(i)} \\ \cos \varphi _{t}^{(i)} \end{pmatrix} \, , \quad i \in \{2, \dots , N\}, $$

while, in the laboratory frame, the positions along the links, each of which is parametrized by an arc-length coordinate $s \in [0, \ell _{i}]$, $i\in \{1,\ldots ,N\}$, are

4.1 $$ \mathbf{x}_{t}^{(1)} (s) = \mathbf{x}_{t} + R_{t} s \tilde{\mathbf{e}}_{t}^{(1)} \, , \qquad \mathbf{x}_{t}^{(i)} (s) = \mathbf{x}_{t} + R_{t} \bigg[ \sum _{j=2}^{i-1} \ell _{j} \tilde{\mathbf{e}}_{t}^{(j)} + s \tilde{\mathbf{e}}_{t}^{(i)} \bigg] \, , \quad i \in \{2,\dots ,N\}, $$

where $t\mapsto \mathbf{x}_{t}$ is the position of the joint between link 1 and link 2 with respect to the origin of the laboratory frame and $t\mapsto R_{t}$ is its orientation. By (4.1) and Resistive Force Theory, we can compute the densities of viscous force and torque $\mathbf{f}_{t}^{(i)}(s)$ and $\boldsymbol{\tau }_{t}^{(i)}(s)$ as in (2.4a)–(2.4c). To derive the equations of motion of the swimmer, both the entries of the grand resistance matrix $\tilde{\mathcal{M}}_{t}$ and the viscous force and torque $\tilde{\mathbf{F}}_{t}^{\mathrm{sh}}$ and $\tilde{\mathbf{T}}_{t}^{\mathrm{sh}}$ due to the shape change must be computed. The block entries of the grand resistance matrix are

4.2 $$ \tilde{K}_{t} :=\sum _{i=1}^{N} \tilde{K}_{t}^{(i)} \, , \qquad \tilde{C}_{t} :=\sum _{i=1}^{N} \tilde{C}_{t}^{(i)} \, , \qquad \tilde{J}_{t} :=\sum _{i=1}^{N} \tilde{J}_{t}^{(i)} $$

where $\tilde{K}_{t}^{(i)}$, $\tilde{C}_{t}^{(i)}$ for $i=1,\ldots ,N$, are given by

4.3 $$ \begin{aligned} \tilde{K}_{t}^{(i)} :=& [(C_{\parallel }-C_{\perp }) \tilde{\mathbf{e}}^{(i)}_{t}\otimes \tilde{\mathbf{e}}^{(i)}_{t}+C_{\perp }I]\ell _{i} \,, \\ \tilde{C}_{t}^{(i)} :=& [(C_{\parallel }-C_{\perp }) \tilde{\mathbf{e}}^{(i)}_{t}\otimes \tilde{\mathbf{e}}^{(i)}_{t}+C_{\perp }I]\ell _{i} \bigg(\sum _{j=2}^{i-1} \ell _{j} \tilde{E}_{t}^{(j)} \bigg) + \frac{\ell _{i}^{2}}{2} C_{\perp }\tilde{E}^{(i)}_{t} \,, \end{aligned} $$

and

$$ \begin{aligned} \tilde{J}_{t}^{(1)} :=& \frac{\ell _{1}^{3}}{3} C_{\perp }\big[ I - \tilde{\mathbf{e}}_{t}^{(1)} \otimes \tilde{\mathbf{e}}_{t}^{(1)}\big] +\ell _{1}C_{\tau }\tilde{\mathbf{e}}_{t}^{(1)} \otimes \tilde{\mathbf{e}}_{t}^{(1)}, \\ \tilde{J}_{t}^{(i)} :=& - \ell _{i} (C_{\parallel }- C_{\perp }) \bigg(\sum _{j=2}^{i-1} \ell _{j} \tilde{E}_{t}^{(j)} \bigg) ( \tilde{\mathbf{e}}_{t}^{(i)} \otimes \tilde{\mathbf{e}}_{t}^{(i)} ) \bigg(\sum _{j=2}^{i-1} \ell _{j} \tilde{E}_{t}^{(j)} \bigg) \\ & - \ell _{i} C_{\perp }\bigg(\sum _{j=2}^{i-1} \ell _{j} \tilde{E}_{t}^{(j)} \bigg)^{2} - \frac{\ell _{i}^{2}}{2} C_{\perp }\tilde{E}_{t}^{(i)} \bigg(\sum _{j=2}^{i-1} \ell _{j} \tilde{E}_{t}^{(j)} \bigg) \\ & - \frac{\ell _{i}^{2}}{2} C_{\perp }\bigg(\sum _{j=2}^{i-1} \ell _{j} \tilde{E}_{t}^{(j)} \bigg) \tilde{E}_{t}^{(i)} + \frac{\ell _{i}^{3}}{3} C_{\perp }\big[ I - \tilde{\mathbf{e}}_{t}^{(i)} \otimes \tilde{\mathbf{e}}_{t}^{(i)}\big] \,,\quad \text{for}\quad i=2, \ldots N. \end{aligned} $$

In the expressions above, the matrices $\tilde{E}_{t}^{(i)}$ represent the vector product $\tilde{\mathbf{e}}_{t}^{(i)}\times $, as in (2.9). The expression of the grand resistance matrix $\tilde{\mathcal{M}}_{t}$ given in (2.11) still holds, using the formulas for the blocks in (4.2) and (4.3). The vectors $\tilde{\mathbf{F}}_{t}^{\mathrm{sh}}$ and $\tilde{\mathbf{T}}_{t}^{\mathrm{sh}}$ are

4.4 $$ \begin{aligned} \tilde{\mathbf{F}}_{t}^{\mathrm{sh}} :=& \sum _{i=2}^{N} \bigg[ [(C_{\parallel }-C_{\perp })\tilde{\mathbf{e}}^{(i)}_{t}\otimes \tilde{\mathbf{e}}^{(i)}_{t}+C_{\perp }I]\ell _{i} \bigg(\sum _{j=2}^{i-1} \ell _{j} \dot{\tilde{\mathbf{e}}}_{t}^{(j)} \bigg) + \frac{\ell _{i}^{2}}{2} C_{\perp }\dot{\tilde{\mathbf{e}}}_{t}^{(i)} \bigg] \\ \tilde{\mathbf{T}}_{t}^{\mathrm{sh}} :=& \sum _{i=2}^{N} \bigg[ \ell _{i} \bigg(\sum _{j=2}^{i-1} \ell _{j} \tilde{E}_{t}^{(j)} \bigg)[(C_{\parallel }-C_{\perp })\tilde{\mathbf{e}}^{(i)}_{t}\otimes \tilde{\mathbf{e}}^{(i)}_{t}+C_{\perp }I] \bigg(\sum _{j=2}^{i-1} \ell _{j} \dot{\tilde{\mathbf{e}}}_{t}^{(j)} \bigg) \\ & + \frac{\ell _{i}^{2}}{2} C_{\perp }\tilde{E}_{t}^{(i)} \bigg(\sum _{j=2}^{i-1} \ell _{j} \dot{\tilde{\mathbf{e}}}_{t}^{(j)} \bigg) + \frac{\ell _{i}^{2}}{2} C_{\perp }\bigg(\sum _{j=2}^{i-1} \ell _{j} \tilde{E}_{t}^{(j)} \bigg) \dot{\tilde{\mathbf{e}}}_{t}^{(i)} + \frac{\ell _{i}^{3}}{3} C_{\perp }\tilde{E}_{t}^{(i)} \dot{\tilde{\mathbf{e}}}_{t}^{(i)} \bigg] \, , \end{aligned} $$

so that, analogously to (2.10), the equations of motion read

4.5 $$ \mathbf{0}= \left ( \textstyle\begin{array}{c} \mathbf{F}_{t} \\ \mathbf{T}_{t} \end{array}\displaystyle \right )=\tilde{\mathcal{M}}_{t}\left [ \textstyle\begin{array}{c@{\quad }c} R_{t}^{-1} &0 \\ 0 &R_{t}^{-1} \end{array}\displaystyle \right ]\left ( \textstyle\begin{array}{c} \dot{\mathbf{x}}_{t} \\ \boldsymbol{\omega }_{t} \end{array}\displaystyle \right )+\left ( \textstyle\begin{array}{c} \tilde{\mathbf{F}}^{\mathrm{sh}}_{t} \\ \tilde{\mathbf{T}}^{\mathrm{sh}}_{t} \end{array}\displaystyle \right ). $$

By recalling that $\tilde{\mathcal{M}}_{t}$ is positive definite, and therefore invertible, (4.5) can be written as

4.6 $$ \begin{aligned} \begin{pmatrix} R_{t}^{-1}\dot{\mathbf{x}}_{t} \\ R_{t}^{-1}\boldsymbol{\omega }_{t} \\ \dot{\varphi }_{t}^{(2)} \\ \dot{\vartheta }_{t}^{(2)} \\ \vdots \\ \dot{\varphi }_{t}^{(N)} \\ \dot{\vartheta }_{t}^{(N)} \end{pmatrix}=\sum _{i=2}^{N} \Big[ & V_{1}^{(i)}\big(\{(\varphi _{t}^{(j)}, \vartheta _{t}^{(j)})\}_{j=2}^{N}\big)u_{1}^{(i)} +V_{2}^{(i)}\big(\{( \varphi _{t}^{(j)},\vartheta _{t}^{(j)})\}_{j=2}^{N}\big)u_{2}^{(i)} \big) \Big], \end{aligned} $$

where, for $i=2,\ldots ,N$, $V_{1}^{(i)}$ and $V_{2}^{(i)}$ are vector fields with $6+2(N-1)=2N+4$ components. The following theorem, whose proof can be obtained by applying classical results from ODE theory (see, *e.g.*, [21]), holds. Notice that the controllability Theorem 4.2 below provides a sufficient condition for existence.

### Theorem 4.1

*Let* $(\bar{x},\bar{R},{\left\{{\bar{\varphi }}^{\left(i\right)},{\bar{\vartheta }}^{\left(i\right)}\right\}}_{i=2}^{N})\in R^{3}\times SO(3)\times {\left((0,\pi )\times (-\pi ,\pi )\right)}^{N-1}$ *be given*. *There exists a unique absolutely continuous solution* $(x_{t},R_{t},{\left\{\varphi_{t}^{\left(i\right)},\vartheta_{t}^{\left(i\right)}\right\}}_{i=2}^{N}):[0,+\infty )\to R^{3}\times SO(3)\times {\left((0,\pi )\times (-\pi ,\pi )\right)}^{N-1}$ *to the Cauchy problem for* (4.6) *with initial condition* $\big (\mathbf{x}_{0}, R_{0}, \{\varphi ^{(i)}_{0},\vartheta ^{(i)}_{0}\}_{i=2}^{N}\big )= \big (\bar{\mathbf{x}},\bar{R},\{\bar{\varphi }^{(i)},\bar{\vartheta }^{(i)} \}_{i=2}^{N}\big )$, *for any controls* $u_{1}^{(i)},u_{2}^{(i)}\in L^{\infty }(0,+\infty )$ *for* $i=2,\ldots ,N$.

### Theorem 4.2

Controllability of the $N$-link

*The* $N$-*link swimmer is controllable if* $C_{\perp}\neq C_{\parallel}$ *and for almost every lengths* $\ell _{i}$ ($i=1,\ldots ,N$) *of the links*.

### Proof

The proof follows the reasoning of that of [17, Theorem 3.1], where it is proved that the controllability of a planar $N$-link swimmer follows from that of a planar Purcell 3-link swimmer. In the present case, from the controllability of the 2-link swimmer in three dimensions, together with the analyticity of the vector fields $\{V_{1}^{(i)},V_{2}^{(i)}\}_{i=2}^{N}$ (introduced in (4.6)) with respect to the $\ell _{i}$’s, we will be able to deduce the controllability of the $N$-link swimmer.

More precisely, by setting $\ell _{1}=\ell _{2}=:L$ and $\ell _{i}=0$ for all $i=3,\ldots ,N$, we reduce the $N$-link swimmer to a 2-link swimmer, which can be described as in Sect. 2. In particular, the equations of motion (4.6) read

$$ \begin{aligned} \begin{pmatrix} R_{t}^{-1}\dot{\mathbf{x}}_{t} \\ R_{t}^{-1}\boldsymbol{\omega }_{t} \\ \dot{\varphi }_{t}^{(2)} \\ \dot{\vartheta }_{t}^{(2)} \\ \vdots \\ \dot{\varphi }_{t}^{(N)} \\ \dot{\vartheta }_{t}^{(N)} \end{pmatrix}= & W_{1}^{(2)}(\varphi _{t}^{(2)},\vartheta _{t}^{(2)})u_{1}^{(2)} +W_{2}^{(2)}(\varphi _{t}^{(2)},\vartheta _{t}^{(2)})u_{2}^{(2)}, \end{aligned} $$

where the first eight components of $W_{1}^{(2)}$ and $W_{2}^{(2)}$, denoted by $w_{1}^{(2)}$ and $w_{2}^{(2)}$, are obtained from those of $V_{1}^{(2)}$ and $V_{2}^{(2)}$, respectively, and the last $2N-4$ components of both $W_{1}^{(2)}$ and $W_{2}^{(2)}$ are zero. Clearly, $w_{1}^{(2)}$ and $w_{2}^{(2)}$ are precisely the $V_{1}$ and $V_{2}$ in (2.14) and (2.15).

By Theorem 3.6, the vector fields $w_{1}^{(2)}$ and $w_{2}^{(2)}$ generate all of the tangent space $\mathrm{se}(3)\times R^{2}$. Indeed, by taking the iterated Lie brackets of $w_{1}^{(2)}$ and $w_{2}^{(2)}$ evaluated at $(e,s^{*})=(e,(\frac{\pi }{2},0))$ as we did in the proof of Theorem 3.6, formula (3.8) holds:

4.7 $$ \delta ^{*}=\det \big(w_{1}^{(2),*}|\cdots |w_{8}^{(2),*}\big)_{( \frac{\pi }{2},0)}= \frac{p(C_{\parallel },C_{\perp }, C_{\tau },L)}{q(C_{\parallel },C_{\perp }, C_{\tau },L)} $$

with the same $p$ and $q$ defined in (3.9), and again it does not vanish if $C_{\perp}\neq C_{\parallel}$. Therefore, the vector fields $w_{1}^{(2),*}$ and $w_{2}^{(2),*}$ generate the 8-dimensional tangent space $\mathrm{se}(3)\times R^{2}$ at the point $(e,s^{*})$. As done for the 2-link swimmer, we argue that from the analyticity of the vector fields and from the Orbit Theorem 3.5, they generate the tangent space $\mathrm{se}(3)\times R^{2}$ at any point $(e,s)$. Thus controllability at any points $(h,s)$ follows for a swimmer with links of lengths $\ell _{1}=\ell _{2}=L$ and $\ell _{i}=0$ for $i>2$. Taking (4.3) and (4.4) into account, it is easy to observe that the vector fields $\{V_{1}^{(i)},V_{2}^{(i)}\}_{i=2}^{N}$ in (4.6) depend analytically on $\ell _{1},\ldots ,\ell _{N}$, so that

4.8 $$ \big(\ell _{1},\ldots ,\ell _{N}\big)\mapsto \delta ^{*}=\det \big( \text{Lie brackets of $V_{1}^{(2)}$, $V_{2}^{(2)}$}\big)_{(\frac{\pi }{2},0)} $$

also does. In particular, (4.7) is the map in (4.8) evaluated at $(L,L,0,\ldots ,0) $. Since (4.7) is different from zero, the analytic map in (4.8) will stay away from zero if $C_{\perp}\neq C_{\parallel}$ and for almost every lengths $\ell _{i}$ of the links. Thus, the vector fields $\{V_{1}^{(i)},V_{2}^{(i)}\}_{i=2}^{N}$ in (4.6) are linearly independent and generate the $(2N+4)$-dimensional Lie algebra in which the equations of motion (4.6) live. Controllability is proved. □

## Optimal Control Problems

In this section we tackle some optimality problems for the 2-link swimmer whose solution we can characterize. The generalizations to the $N$-link swimmer are easily deduced by consideration of some geometric constraints, such as non interpenetration. Recalling the notation of Sect. 3.1, given $(g,s)\in G\times \mathcal{S}$ the state variable, and $u\in U$, where $U\subset R^{n}$ is the compact set of controls, with the origin belonging to the interior of $U$, solving a generic control problem for (3.1) amounts to minimizing the time integral of a Lagrangian $L:G\times S\times U\to R^{+}$ under suitable constraints, namely

5.1 $$ \textstyle\begin{cases} \displaystyle \inf \bigg\{ \int _{0}^{t_{f}} \mathscr{L}(g_{t},s_{t},u_{t}) \,\mathrm{d}t \bigg\} , \\ \text{$(g_{t},s_{t},u_{t})\in G\times \mathcal{S}\times U$ for every $t\in [0,t_{f}]$,} \\ \text{(3.1) holds for every $t\in [0,t_{f}]$,} \\ (g_{0},s_{0})=(\bar{g}^{0},\bar{s}^{0}), (g_{t_{f}},s_{t_{f}})=(\bar{g}^{1},\bar{s}^{1}), \end{cases} $$

where $t_{f}>0$ is a final time, and $(\bar{g}^{0},\bar{s}^{0})$ and $(\bar{g}^{1},\bar{s}^{1})$ are prescribed initial and final configurations of the system, respectively.

Recalling (3.5), for the 2-link swimmer we have $G=SE(3)$ and $\mathcal{S}=(0,\pi )\times (-\pi ,\pi )$. Finally, $u_{t}=(u_{1,t},u_{2,t}):[0,t_{f}]\to U\subset R^{2}$, with $u_{1}$ and $u_{2}$ introduced in (2.12). Therefore, we can recast the optimal control problem (5.1) for the 2-link swimmer as

5.2 $$ \textstyle\begin{cases} \displaystyle \inf \bigg\{ \int _{0}^{t_{f}} \mathscr{L}(g_{t}, \varphi _{t},\vartheta _{t},u_{1,t},u_{2,t})\,\mathrm{d}t \bigg\} , \\ \text{$(u_{1,t},u_{2,t})\in U$ for every $t\in [0,t_{f}]$,} \\ \text{(3.7) holds for every $t\in [0,t_{f}]$,} \\ (g_{0},\varphi _{0},\vartheta _{0})=(\bar{g}^{0},\bar{\varphi}^{0},\bar{\vartheta}^{0}), (g_{t_{f}},\varphi _{t_{f}},\vartheta _{t_{f}})=(\bar{g}^{1},\bar{\varphi}^{1},\bar{\vartheta}^{1}). \end{cases} $$

We can state a general result for (5.2).

### Theorem 5.1

*Let* $L:SE(3)\times (0,\pi )\times (-\pi ,\pi )\times U\to R^{+}$ *be smooth*. *Then there exists a solution to the optimal control problem* (5.2), *namely*, *there exist an absolutely continuous trajectory* $\hat{g}\colon [0,t_{f}]\to SE(3)$, *absolutely continuous shape changes* $(\hat{\varphi },\hat{\vartheta })\colon [0,t_{f}]\to (0,\pi )\times (- \pi ,\pi )$, *and bounded controls* $(\hat{u}_{1},\hat{u}_{2})\colon [0,t_{f}]\to U$ *such that*

5.3 $$ \inf \bigg\{ \int _{0}^{t_{f}} \mathscr{L}(g_{t},\varphi _{t}, \vartheta _{t},u_{1,t},u_{2,t})\,\mathrm{d}t \bigg\} =\int _{0}^{t_{f}} \mathscr{L}(\hat{g}_{t},\hat{\varphi }_{t},\hat{\vartheta }_{t},\hat{u}_{1,t}, \hat{u}_{2,t})\,\mathrm{d}t, $$

$(\hat{u}_{1,t},\hat{u}_{2,t})\in U$ *for every* $t\in [0,t_{f}]$, (3.7) *holds for every* $t\in [0,t_{f}]$, *and* $(\hat{g}_{0},\hat{\varphi}_{0},\hat{\vartheta}_{0})=(\bar{g}^{0},\bar{\varphi}^{0},\bar{\vartheta}^{0})$, $(\hat{g}_{t_{f}},\hat{\varphi}_{t_{f}},\hat{\vartheta}_{t_{f}})=(\bar{g}^{1},\bar{\varphi}^{1},\bar{\vartheta}^{1})$ *where* $(\bar{g}^{0},\bar{\varphi}^{0},\bar{\vartheta}^{0}), (\bar{g}^{1},\bar{\varphi}^{1},\bar{\vartheta}^{1}) \in SE(3)\times (0,\pi )\times (-\pi ,\pi )$ *are given*.

### Proof

By Theorem 3.6, the system is controllable with bounded controls, therefore the set of controls on which the infimum in (5.3) is taken is not empty. Since the set $U$ of controls is compact, since the dynamics (3.7) is linear in the controls, and since the vector fields $V_{1}$ and $V_{2}$ in (2.14) and (2.15) are analytic, the hypotheses of [1, Theorem 10.3] are satisfied, yielding the compactness of the set of reachable points starting from $(\bar{g}^{0},\bar{\varphi}^{0},\bar{\vartheta}^{0})$. Therefore, the optimal control problem (5.2) has a solution. □

We now discuss the solution to some specific optimal control problems, which can be directly obtained from Theorem 5.1, by making suitable choices of the Lagrangian ℒ.

The *minimal time optimal control problem* for the 2-link swimmer can be written as: given $(\bar{g}^{0},\bar{\varphi}^{0},\bar{\vartheta}^{0}),(\bar{g}^{1},\bar{\varphi}^{1},\bar{\vartheta}^{1})\in SE(3)\times (0,\pi )\times (-\pi ,\pi ) $, solve

5.4 $$ \textstyle\begin{cases} \displaystyle \inf t_{f}, \\ \text{$(u_{1,t},u_{2,t})\in U$ for every $t\in [0,t_{f}]$,} \\ \text{(3.7) holds for every $t\in [0,t_{f}]$,} \\ (g_{0},\varphi _{0},\vartheta _{0})=(\bar{g}^{0},\bar{\varphi}^{0},\bar{\vartheta}^{0}), (g_{t_{f}},\varphi _{t_{f}},\vartheta _{t_{f}})=(\bar{g}^{1},\bar{\varphi}^{1},\bar{\vartheta}^{1}). \end{cases} $$

The *optimal control for the power expended* is the following. Let us recall that, for a motion defined on the fixed time interval $[0,t_{f}]$, the power expended is defined as the scalar product of the force against the velocity, namely

$$\mathcal{P}:=\sum _{i=1}^{N} \int _{0}^{t_{f}} \int _{0}^{ \ell _{i}} \Big[\left \langle \mathbf{f}_{t}^{(i)}(s), \dot{\mathbf{x}}_{t}^{(i)}(s)\right \rangle +\left \langle \boldsymbol{\tau }_{t}^{(i)}(s),\boldsymbol{\omega }_{t}\right \rangle \Big]\mathrm{d}s\mathrm{d}t. $$

Taking (2.5) and (2.6) into account, the power for the 2-link swimmer analysed in previous Sects. 2 and 3 ($N=2$ and $\ell _{i}=L$ for $i=1,2$) reads

$$ \begin{aligned} \mathcal{P}= & \int _{0}^{t_{f}} \int _{0}^{L} \Big[ \left \langle \mathbf{f}_{t}^{(1)}(s),\dot{\mathbf{x}}_{t}^{(1)}(s) \right \rangle +\left \langle \mathbf{f}_{t}^{(2)}(s), \dot{\mathbf{x}}_{t}^{(2)}(s)\right \rangle +\left \langle \boldsymbol{\tau }_{t}^{(1)}(s)+\boldsymbol{\tau }_{t}^{(2)}(s), \boldsymbol{\omega }_{t}\right \rangle \Big] \mathrm{d}s\mathrm{d}t \\ = & \int _{0}^{t_{f}} \bigg[ \frac{L^{3} C_{\perp }\big(4C_{\parallel }+ C_{\perp }+(4C_{\parallel }-C_{\perp })\cos \varphi _{t}\big)}{24\big(C_{\parallel }+C_{\perp }+(C_{\parallel }-C_{\perp }) \cos \varphi _{t}\big)} \dot{\varphi }_{t}^{2} \\ & \phantom{\int _{0}^{t_{f}}\bigg[ } + \frac{12 C_{\tau }^{2} C_{\perp }L^{3} \sin ^{2}\varphi _{t} (5 (\cos (2 \varphi _{t} )+3)-12 \cos \varphi _{t})}{\left (-36 C_{\tau }\cos \varphi _{t}+45 C_{\tau }+\cos (2 \varphi _{t} ) \left (15 C_{\tau }-2 C_{\perp }L^{2}\right )+2 C_{\perp }L^{2}\right )^{2}} \dot{\vartheta }^{2}\bigg]\,\mathrm{d}t. \end{aligned} $$

The power expended $\mathcal{P}$ is expected to be a function of the shape parameters and of their velocities and in particular it is quadratic in the velocities.

Recalling that we posed $u_{1}=\dot{\varphi }$ and $u_{2}=\dot{\vartheta }$ (see (2.14) and (2.15)), the optimal control problem for the power expended can be cast in the form (5.2) by taking

5.5 $$\begin{aligned} &\mathscr{L}(g,\varphi ,\vartheta ,u_{1},u_{2})=\mathscr{L}_{ \mathcal{P}}(\varphi ,u_{1},u_{2}) \\ &\quad :=\frac{L^{3} C_{\perp }\big(4C_{\parallel }+ C_{\perp }+(4C_{\parallel }-C_{\perp })\cos \varphi \big)}{24\big(C_{\parallel }+C_{\perp }+(C_{\parallel }-C_{\perp }) \cos \varphi \big)} u_{1}^{2} \\ &\qquad + \frac{12 C_{\tau }^{2} C_{\perp }L^{3} \sin ^{2}\varphi (5 (\cos (2 \varphi )+3)-12 \cos (\varphi ))}{\left (-36 C_{\tau }\cos \varphi +45 C_{\tau }+\cos (2 \varphi ) \left (15 C_{\tau }-2 C_{\perp }L^{2}\right )+2 C_{\perp }L^{2}\right )^{2}}u_{2}^{2} \,, \end{aligned}$$

namely

5.6 $$ \textstyle\begin{cases} \displaystyle \inf \bigg\{ \int _{0}^{t_{f}} \mathscr{L}_{\mathcal {P}}(\varphi _{t},u_{1,t},u_{2,t})\,\mathrm {d}t \bigg\} , \\ \text{$(u_{1,t},u_{2,t})\in U$ for every $t\in [0,t_{f}]$,} \\ \text{(3.7) holds for every $t\in [0,t_{f}]$,} \\ (g_{0},\varphi _{0},\vartheta _{0})=(\bar{g}^{0},\bar{\varphi}^{0},\bar{\vartheta}^{0}), (g_{t_{f}},\varphi _{t_{f}},\vartheta _{t_{f}})=(\bar{g}^{1},\bar{\varphi}^{1},\bar{\vartheta}^{1}). \end{cases} $$

Theorem 5.1 has the following immediate corollary.

### Corollary 5.2

*For any* $(\bar{g}^{0},\bar{\varphi}^{0},\bar{\vartheta}^{0}),(\bar{g}^{1},\bar{\varphi}^{1},\bar{\vartheta}^{1})\in SE(3)\times (0,\pi )\times (-\pi ,\pi ) $, *there exists a solution to problems* (5.4) *and* (5.6).

### Proof

It is enough to apply Theorem 5.1 with $\mathscr{L}\equiv 1$ to obtain the existence of a solution to (5.4) and $\mathscr{L}=\mathscr{L}_{\mathcal{P}}$ to obtain the existence of a solution to (5.6). □

Since we have proved that system (3.7) is controllable, the characterization of the controls can be obtained if no singular arcs occur in the dynamics (see [40, Definition 5.3.1] for the notion of singular arc). The minimal time optimal control problem and the optimal control problem for the power expendedare now analyzed.

### Theorem 5.3

*Assume that no singular arcs occur in the dynamics* (3.7). *Then*, *for any* $(\bar{g}^{0},\bar{\varphi}^{0},\bar{\vartheta}^{0}),(\bar{g}^{1},\bar{\varphi}^{1},\bar{\vartheta}^{1})\in SE(3)\times (0,\pi )\times (-\pi ,\pi ) $, *there exists a solution to*

5.7 $$ \textstyle\begin{cases} \inf t_{f}, \\ \textit{(3.7) holds for every $t\in [0,t_{f}]$,} \\ (u_{1,t},u_{2,t})\in U,\;\textit{for every $t\in [0,t_{f}]$,} \\ (g_{0},\varphi _{0},\vartheta _{0})=(\bar{g}^{0},\bar{\varphi}^{0},\bar{\vartheta}^{0}), (g_{t_{f}},\varphi _{t_{f}},\vartheta _{t_{f}})=(\bar{g}^{1},\bar{\varphi}^{1},\bar{\vartheta}^{1}), \end{cases} $$

*namely there exist* ${\hat{t}}_{f}\in R$, *absolutely continuous functions* $(\hat{g},\hat{\varphi},\hat{\vartheta})\colon [0,\hat{t}_{f}]\to SE(3)\times (0,\pi )\times (-\pi ,\pi )$, *and bounded controls* $(\hat{u}_{1},\hat{u}_{2})\colon [0,\hat{t}_{f}]\to U$ *of bang*-*bang type such that the infimum in* (5.7) *is attained at* $\hat{t}_{f}$.

### Proof

Controllability proved in Theorem 3.6 and Filippov’s Theorem grant existence of a solution, see [1]. Recalling the notation $z=(g,\varphi ,\vartheta )\in SE(3)\times (0,\pi )\times (-\pi ,\pi )\subset R^{8}$ of Sect. 3 and denoting $u=(u_{1},u_{2})$, since (3.7) is linear in the controls $u$, it can be written as $\dot{\mathbf{z}}=\mathcal{V}(\mathbf{z})u$, for a certain $8\times 2$ matrix $\mathcal{V}\colon SE(3)\times (0,\pi )\times (-\pi ,\pi )\to (SE(3) \times (0,\pi )\times (-\pi ,\pi ))^{2}$.

To characterize the controls, we apply the Pontryagin Maximum Principle (PMP for short; see [1]): let us introduce the Hamiltonian $H:(SE(3)\times (0,\pi )\times (-\pi ,\pi ))\times R^{2}\times {\left(SE(3)\times (0,\pi )\times (-\pi ,\pi )\right)}^{*}\to R$ defined by

$$ \mathscr{H}(\mathbf{z}_{t},u_{t},\boldsymbol{\lambda }_{t}):=\mathscr{L}(\mathbf{z}_{t},u_{t})+\langle \boldsymbol{\lambda }_{t}, \dot{\mathbf{z}}_{t}\rangle =1+\langle \boldsymbol{\Lambda }_{t},u_{t} \rangle ,\quad t\in [0,\hat{t}_{f}],$$

where $\boldsymbol{\Lambda }_{t,j}=\sum _{i=1}^{8} \lambda _{t,i}\mathcal{V}_{ij}( \mathbf{z}_{t})$, and we notice that it retains the linearity in the controls $u=(u_{1},u_{2})$. PMP prescribes that, if a pair $(\hat{\mathbf{z}},\hat{u})$ is a solution to (5.7), then there exists an absolutely continuous function $\hat{\boldsymbol{\lambda }}\colon [0,t_{f}]\to (SE(3)\times (0,\pi ) \times (-\pi ,\pi ))^{*}$, not identically zero, such that

5.8 $$ \textstyle\begin{cases} \dot{\hat{\mathbf{z}}}=\nabla _{\boldsymbol{\lambda }} \mathscr{H}( \hat{\mathbf{z}},\hat{u},\hat{\boldsymbol{\lambda }}), \\ \dot{\hat{\boldsymbol{\lambda }}}=-\nabla _{\mathbf{z}} \mathscr{H}( \hat{\mathbf{z}},\hat{u},\hat{\boldsymbol{\lambda }}), \\ \hat{u}\in \operatorname*{argmin}\{\mathscr{H}(\hat{\mathbf{z}},u, \hat{\boldsymbol{\lambda }}): u\in U\}. \end{cases} $$

(Notice that the first equation in (5.8) is indeed (3.7)). Thanks to the linearity of the Hamiltonian with respect to the controls, the optimal controls $\hat{u}$ belong to the boundary of $U$, namely they are of bang-bang type. □

### Theorem 5.4

*Assume that no singular arcs occur in the dynamics* (3.7). *Given* $t_{f}>0$, *for any* $(\bar{g}^{0},\bar{\varphi}^{0},\bar{\vartheta}^{0}),(\bar{g}^{1},\bar{\varphi}^{1},\bar{\vartheta}^{1})\in SE(3)\times (0,\pi )\times (-\pi ,\pi ) $, *there exists a solution to*

5.9 $$ \textstyle\begin{cases} \displaystyle \inf \bigg\{ \int _{0}^{t_{f}} \mathscr{L}_{\mathcal{P}}( \varphi _{t},u_{1,t},u_{2,t})\,\mathrm{d}t \bigg\} , \\ \textit{$(u_{1,t},u_{2,t})\in U$ for every $t\in [0,t_{f}]$,} \\ \textit{(3.7) holds for every $t\in [0,t_{f}]$,} \\ (g_{0},\varphi _{0},\vartheta _{0})=(\bar{g}^{0},\bar{\varphi}^{0},\bar{\vartheta}^{0}), (g_{t_{f}},\varphi _{t_{f}},\vartheta _{t_{f}})=(\bar{g}^{1},\bar{\varphi}^{1},\bar{\vartheta}^{1}), \\ \end{cases} $$

*namely there exist an absolutely continuous trajectory* $\hat{g}\colon [0,t_{f}]\to SE(3)$, *absolutely continuous shape changes* $(\hat{\varphi },\hat{\vartheta })\colon [0,t_{f}]\to (0,\pi )\times (- \pi ,\pi )$, *and bounded controls* $(\hat{u}_{1},\hat{u}_{2})\colon [0,t_{f}]\to U$, *either continuous or of bang*-*bang type*, *such that*

$$\inf \bigg\{ \int _{0}^{t_{f}} \mathscr{L}_{\mathcal{P}}(\varphi _{t},u_{1,t},u_{2,}) \,\mathrm{d}t \bigg\} =\int _{0}^{t_{f}} \mathscr{L}_{\mathcal{P}}( \hat{\varphi }_{t},\hat{u}_{1,t},\hat{u}_{2,t})\,\mathrm{d}t. $$

### Proof

The controllability proved in Theorem 3.6 and Filippov’s Theorem grant existence of a solution, see [1]. The regularity of $\hat{u}_{1}$ and $\hat{u}_{2}$ is a consequence of a standard application of PMP which can be used in the same fashion as in the proof of Theorem 5.3, with the Hamiltonian given by

$$ \mathscr{H}(\mathbf{z}_{t},u_{t},\boldsymbol{\lambda }_{t}):=\mathscr{L}_{\mathcal{P}}(\varphi _{t},u_{1,t},u_{2,t})+\langle \boldsymbol{\lambda }_{t},\dot{\mathbf{z}}_{t}\rangle .$$

The optimal controls $t\mapsto (\hat{u}_{1,t},\hat{u}_{2,t})$ are continuous if the stationary point belongs to $U$ for all $t\in [0,t_{f}]$; otherwise, $\hat{u}_{1}$ and $\hat{u}_{2}$ are of bang-bang type. □

## Conclusions and Outlook

In this paper we studied the dynamics, controllability, and optimal control problems for a 2-link swimmer capable of performing fully three-dimensional shape changes. In Sect. 2, we described the configuration and shape of the swimmer and derived the equations of motion of the 2-link swimmer in a low Reynolds number flow by means of Resistive Force Theory and enforcing the so-called *self-propulsion constraint* (setting the viscous force and torque equal to zero, see (2.10) and (2.12)). Theorem 2.2 states the existence and uniqueness of the solution to the equations of motion (2.12). It is derived directly from Theorem 3.6, which is the main result of the paper and the core of Sect. 3. The proof of Theorem 3.6 is achieved by applying techniques from Geometric Control Theory.

In Sect. 4 we extended the results to the case of a general, fully three-dimensional $N$-link swimmer, exploiting the analyticity of the vector fields governing the dynamics. Finally, in Sect. 5, we addressed two specific optimal control problems for the 2-link swimmer, namely the minimal time optimal control problem and the minimization of the power expended. Both problems have an independent interest and find their relevance in the design of artificial micro-devices which mimic the motion of natural micro-organisms.

The results obtained in this paper focus on the self-propulsion case, as it is the first step towards the design of self-propelling micro-robots. Nonetheless, it can be interesting for the applications, and object of future work, to extend the study to externally driven micro-swimmers. This direction has already been pursued in the case of two-dimensional magneto-elastic swimmers: in [3, 4] a planar $N$-link is studied, showing that it can achieve a non-zero net displacement when actuated by a sinusoidal external magnetic field; in [18] local controllability of a 2-link magneto-elastic swimmer is proved, wheres in [15] the actuation of a three-dimensional $N$-link swimmer by an external magnetic field is studied. Finally, we mention that the case of a multi-flagellar swimmer is studied in [41]. Imposing an external actuating field on the one hand has the benefit of *helping* the swimmer to move and simplifying its design from the engineering point of view, while on the other hand makes the problem more challenging from the mathematical point of view.

## Footnotes

1 $(\mathbf{a}\otimes \mathbf{b})\mathbf{v}=(\mathbf{b}\cdot \mathbf{v})\mathbf{a}$; $(\boldsymbol{\omega }\times \mathbf{e})\cdot \mathbf{e}=0$.
